# An inter-rater reliability study of a modified version of SATS as a prehospital triage tool

**DOI:** 10.1186/s13049-026-01648-8

**Published:** 2026-06-12

**Authors:** Louise Deshayes, Magnus Andersson Hagiwara, Andreas Wladis, Denise Bäckström

**Affiliations:** 1 Department of Biomedical and Clinical Sciences (BKV), Faculty of Medicine and Health Sciences, Linköping University, Linköping, 581 83 Sweden; 2 Centre for Research and Development (CFUG), Uppsala University/Gävleborg Region, Gävle, Sweden; 3 Centre for Prehospital Research, Faculty of Caring Science, Work Life and Social Welfare, University of Borås, Borås, SE-501 90 Sweden

**Keywords:** SATS, Triage, Reliability, EMS, Prehospital care

## Abstract

**Background:**

Triage systems play an essential role in allocating patients to an appropriate level of care. Several triage systems are used worldwide, all of which require scientific evaluation of their reliability and validity. Evidence regarding prehospital triage systems remains limited. The South African Triage Scale (SATS) has been adapted for local use in several regions, but modified versions require independent evaluation. The objective of this study was to examine the reliability of a modified version of the triage system, SATS, when it is used in the emergency medical services (EMS) in a region in Sweden. The study also investigated whether triage performance differs among registered nurses with varying experience and competence levels.

**Methods:**

This prospective cross-sectional inter-rater reliability study included 34 registered nurses (RNs) working in the regional EMS. The participants independently triaged 30 prehospital case vignettes via a digital questionnaire, resulting in 1,010 triage assessments. Descriptive data were used. Inter-rater reliability was assessed via Krippendorff’s alpha (α) and percentage agreement. Mixed effects logistic regression was performed to explore associations between triage assignment and RN characteristics, including gender, years of experience and specialist education.

**Results:**

This study indicated insufficient inter-rater agreement when a modified version of SATS was used in a prehospital setting. Krippendorff’s α was 0.60, suggesting poor agreement when triage levels were treated as ordinal data. Full agreement was observed in three case vignettes, whereas agreement across the remaining cases ranged from 32% to 94%. Manual up-triage occurred in 25 of the 30 vignettes, with frequencies ranging from 6% to 74%. No associations were found between assigned triage level and gender, years of EMS experience or specialist education.

**Conclusion:**

This modified version of SATS demonstrated low inter-rater reliability when applied in a prehospital setting. Agreement varied substantially across patient scenarios, particularly in cases characterized by nonspecific symptoms and near-normal vital signs. These findings underscore the importance of systematic evaluation of modified triage systems to support consistent decision-making and patient safety in prehospital care.

**Supplementary Information:**

The online version contains supplementary material available at 10.1186/s13049-026-01648-8.

## Background

Emergency medical services (EMSs) use triage when attending patients to determine the speed of transport and choice of hospital destination for initial treatment, as well as the level of acuity, and differentiating acute patients from non-acute patients. At the emergency department (ED), the triage outcome determines the time and sequence in which the patient should then be seen by a physician. The objective of triage is to identify and prioritize patients with critical time-sensitive conditions and needs. Early triage systems were primarily trauma-based but have been developed to manage the full spectrum of clinical presentations [1]. Nevertheless, studies involving prehospital triage have focused primarily on trauma patients, and more studies focusing on non-trauma patients are needed [2, 3]. Internationally, several triage systems are used within the EMS [3] but are less commonly evaluated than triage in the ED [4, 5]. The prehospital EMS environment presents distinct challenges, including limited and sometimes incomplete patient information [6], unpredictable conditions that vary by location and time of day [7, 8], constrained access to advanced diagnostic resources, such as laboratory testing and imaging modalities, which are not routinely available in the EMS [9, 10]. Also, immediate collegial support may be limited [11]. Consequently, prehospital triage cannot be assumed to perform similarly to triage conducted in the ED and should be evaluated separately.

Evaluating the performance of triage systems is essential to ensure that they can accurately differentiate between high- and low-acuity patients. However, triage systems are inherently difficult to compare [3] because they vary in their construction, use different criteria, and may fail to identify certain serious conditions—particularly when symptoms are nonspecific or when physiological responses vary, as is common among older adults [12, 13]. Time-critical conditions such as acute coronary syndrome, sepsis, and stroke may also present atypically, posing additional challenges for prehospital assessment [14–16]. Reliability is a fundamental aspect of validating a triage system [17]; however, most reliability studies have been conducted in ED settings [18–22]. Although some prehospital studies exist [23–25], research remains limited, with inter-rater reliability (IRR) being the most frequently applied measure.

The South African Triage Scale (SATS) was originally developed for use in prehospital and emergency care settings in South Africa [25, 26]. It is widely implemented in low-resource environments worldwide [27], but its use in high-income countries remains rare. SATS is a nonproprietary triage system distributed under a Creative Commons license, allowing local adaptation and modification to meet regional clinical needs [28]. In Sweden, modified versions of SATS is used in several emergency departments, often with local adjustments. In the prehospital setting, two Swedish regions use SATS-derived systems; however, these differ in structure and algorithm [29]. The modified version of SATS examined in this study is currently implemented prehospital in only one region [30] since its introduction in 2018.

Since its introduction, the system has been subsequently adapted through collaboration between specialist physicians from multiple disciplines, influenced by SATS Norway [31]. The resulting modified version differs substantially from the original SATS developed by the South African Triage Group in 2004 [26], including partial modifications to score calculations of vital signs and revised clinical discriminators. The modifications were intended to align the system with local clinical demands, such as Swedish national guidelines, but have not yet been evaluated scientifically. To date, only one study has assessed the reliability of SATS in a prehospital setting [32]. However, the study evaluated the original SATS version in a low-income setting, limiting comparability to the present context.

The Norwegian adaptation of SATS, which structurally has the greatest resemblance to the modified version presented in this study, has shown good sensitivity for identifying patients with serious outcomes, as well as acceptable levels of under- and overtriage [33]. However, data on its reliability is currently lacking. Given that triage systems serve as critical decision-support tools, it is essential that they demonstrate adequate reliability to ensure patient safety. In advancing evidence-based prehospital practice, there is a clear knowledge gap regarding the reliability of modified versions of the SATS when used by EMS clinicians.

The objective of this study is to examine the reliability of a modified version of the triage system SATS when it is used in the EMS in a region in Sweden. Additionally, the study seeks to investigate whether triage performance differs among RNs with varying experience and competence levels.

## Methods

### Study design

This study was a prospective cross-sectional inter-rater reliability (IRR) study based on 30 case vignettes. The study followed the Guidelines for Reporting Reliability and Agreement Studies (GRAAS) [34].

### Study setting and population

The study was conducted in a centrally located Swedish region with approximately 286,000 inhabitants, covering both urban and rural areas, including sparsely populated districts located far from the nearest hospital. The region covers 18,000 km² (population density 16 inhabitants/km²). Approximately half of the population is under 45 years of age, and life expectancy is 83.9 years for women and 80.5 years for men [35].

The region has three hospitals, two of which provide comprehensive emergency care, while the third only mainly receives patients with acute medical conditions. The regional EMS consists of approximately 25 ambulance units, including two single-responder units, stationed across 11 sites. In 2024, 44,218 ambulance assignments were carried out.

The EMS workforce comprises approximately 250 employees, the majority of whom are registered nurses (RNs). Ambulances are always staffed by at least one RN and may also include emergency medical technicians (EMTs) and specialized nurses. Specialized nurses hold master’s degrees with a focus on either prehospital emergency care or other relevant clinical areas. EMTs have assistant nurse training combined with supplementary education in prehospital care.

Within each ambulance team, the RN with the highest level of formal qualification assumes clinical responsibility. This includes the overall responsibility for patient assessment, clinical decision-making, and determining the final triage level. Treatment follows regional clinical guidelines, which outline assessment and management pathways for a range of emergency presentations [36]. Ambulances are equipped with basic diagnostic and monitoring equipment, including blood pressure cuff, stethoscope, pulse oximeter, thermometer, electrocardiogram (ECG) and blood glucose meter.

### Participants

All clinically active RNs employed within the regional EMS (*n* = 223) were eligible for participation. Recruitment was conducted through email invitations, informational posters displayed at all ambulance stations, and verbal announcements during workplace meetings. Participation was voluntary, and all eligible RNs were invited regardless of years of experience or educational background.

### Case vignettes

The case vignettes were developed to reflect the most common prehospital presenting complaints in Sweden [37]. They were designed to include a broad range of clinical presentations and to cover all four SATS acuity levels (green, yellow, orange, and red), as well as several discriminators relevant to the triage process. The case vignettes describe adult patients contacting EMS, including information on presenting complaints, background, and clinical findings at assessment. The vignettes were constructed by LD based on literature and clinical experience. Three co-authors (DB, AW, and MH), all with extensive backgrounds in prehospital and emergency care, independently reviewed the vignettes and confirmed that the vignettes were realistic and clinically meaningful.

The vignettes were pilot tested by two specialist nurses with extensive experience in emergency care and triage. Their feedback led to minor clarifications, and the final vignettes were considered authentic and suitable for evaluating inter-rater reliability. These specialist nurses did not participate in the actual study. The supplementary materials include the full set of vignettes (Additional file 1).

### Triage instrument

**Fig. 1:**
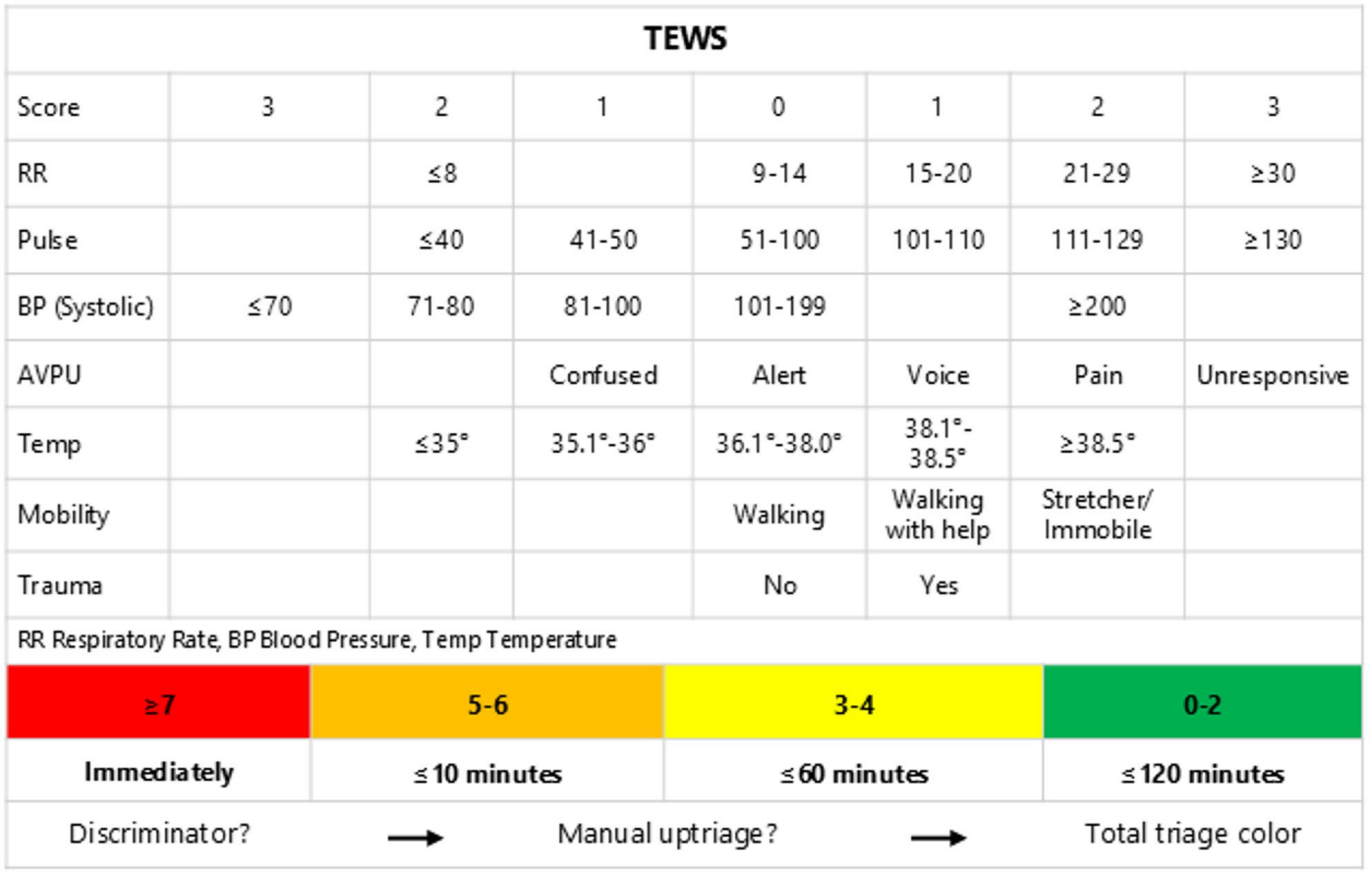
Description of triage by SATS as used in the study and vital signs included in the Triage early warning score (TEWS) including waiting time to physician

Within the EMS, SATS is a four-tiered triage system in which acuity levels are represented by color codes corresponding to clinical urgency and the recommended waiting time (Fig. 1). The system consists of two complementary components. The first is the Triage Early Warning Score (TEWS), a physiological scoring system that combines vital signs into a cumulative score, with defined score ranges corresponding to specific triage levels. TEWS includes two parameters that rely on clinical judgment rather than fixed physiological thresholds: mobility assessment and a trauma factor. The mobility item assigns a higher score to patients with impaired ability to ambulate, whereas the trauma factor increases the score for suspected trauma cases. Because these components require evaluator interpretation, they may introduce additional inter-rater variability. The second component is a set of clinical discriminators, which represent key symptoms, signs, or presentations that indicate acuity independent of vital sign derangements (Additional file 2). If a discriminator suggests a higher level of urgency than the TEWS score does, the discriminator determines the final triage category.

In addition, the assessing clinician may override the algorithm by manual up-triage when the patient’s condition is perceived as more severe than captured by the TEWS or the available discriminators. This feature is intended to ensure patient safety in atypical presentations but may also introduce variation in inter-rater assessments.

### Data collection

Data were collected over an eight-week period in the autumn of 2025. An email invitation containing study information was distributed to all eligible RNs working in the regional EMS. RNs who expressed interest in participating were subsequently provided with a link to a digital questionnaire consisting of 30 fictive case vignettes, which could be completed at any time and location of the participants’ choice. The participants were able to save their responses and complete the questionnaire in multiple sessions. In each vignette, vital signs were presented, including airway status, respiratory rate, pulse and heart rate when necessary, blood pressure, mental status, temperature, blood glucose, and pain assessment when applicable. This resulted in a baseline TEWS score for each vignette. Participants were instructed to calculate the total TEWS score by incorporating a trauma factor and an appropriate mobility score based on the descriptive information provided in each vignette (Additional file 3). They were then asked to add a discriminator if appropriate. Thereafter, a final triage color was assigned to each vignette, based on total TEWS score and potential discriminator. Finally, participants could manually uptriage each patient if they deemed it necessary. The full set of questions is available in Additional file 3. Completion of the 30 vignettes required approximately 70 min. They were explicitly instructed to complete the questionnaire independently, without consulting colleagues; however, they were permitted to use clinical guidelines and conduct internet searches, reflecting resources available in real-life prehospital practice. They were further informed that there were no predefined correct answers to encourage triage decisions based on their own clinical judgment. A reminder was sent to all invited RNs two weeks after the initial invitation. Two additional reminders were then sent exclusively to those who had indicated interest but had not yet completed the questionnaire. No identifying information was collected.

### Data analysis

The reported questionnaire data were entered into Microsoft Excel and subsequently imported into R version 4.4.3 (R Core Team, Vienna, Austria) for statistical analysis. Descriptive statistics were used to summarize the demographic and professional characteristics of the participating RNs.

Total TEWS scores were corrected in cases of obvious miscalculation, as the electronic health record system automatically calculates the TEWS score in clinical practice.

Inter-rater reliability was assessed via Krippendorff´s alpha (α). The statistic α was applied to ordinal triage ratings, as it accommodates multiple raters, varying numbers of observations per rater, and missing data [38]. Agreement was evaluated for triage outcomes both including and excluding manual uptriage. The triage outcome was additionally dichotomized into high- and low-acuity (orange/red vs. green/yellow) categories to reflect the clinical distinction between urgent and non-urgent patients, thereby improving interpretability of agreement at a decision-critical threshold. Krippendorff´s alpha was calculated for the dichotomized data using a nominal scale.

The values range from − 1 to 1, where 1.0 indicates perfect agreement. According to the recommended thresholds, values ≥ 0.80 denote reliable agreement suitable for drawing conclusions, values between 0.67 and 0.79 indicate moderate agreement requiring cautious interpretation, and values < 0.67 reflect poor agreement [39]. The percentage agreement was also calculated to provide a complementary, unadjusted measure of concordance across the raters.

To assess the impact of variability in mobility assessment on final triage categorization, individual TEWS scores were recalculated by combining the baseline TEWS score with the mobility score assigned by each RN. The resulting total TEWS (baseline TEWS + mobility score) scores were subsequently dichotomized into low-acuity (green/yellow) and high-acuity (orange/red). For each vignette, the distribution of high- versus low-acuity classifications across raters was examined. Vignettes were classified as inconsistent if both high- and low-acuity categorizations were present among raters. To further examine the degree of disagreement, the proportion of raters assigning high-acuity was calculated for each vignette classified as inconsistent.

In addition, a mixed-effects logistic regression model was used to examine the association between RN characteristics and triage levels, where red or orange triage was considered high-acuity, and green or yellow triage was considered low-acuity. The model included fixed effects for specialist qualifications, clinical experience in the EMS, and gender, and random intercepts for nurses and case vignettes to account for the cross-classified data structure.

## Results

A total of 34 RNs completed the study. The demographic and professional characteristics of the participants are presented in Table 1. All the participants had received SATS training. Those employed at the time of implementation completed a one-day introductory course, whereas participants employed thereafter completed a two-hour training session. Participants could report more than one type of SATS training, and 76% reported completion of the annually updated web-based SATS course. 41% of the participants reported receiving peer-led training.

**Table 1:** Demographics and professional characteristics of the participating RNs

Variable	*n* (%) or Mean ± SD	Range/Categories
**Sex**		
Female	24 (70.6)	
Male	10 (29.4)	
**Specialist qualification**		
Prehospital emergency care	11 (32.4)	
Anaesthesia	2 (5.9)	
Emergency nursing	1 (2.9)	
None	18 (52.9)	
Other	2 (5.9)	
**SATS training combinations**		
Instructor-led training (2 h) + web-based SATS course + colleague training	9 (26.0)	
One-day introductory course + web-based SATS course	7 (21.0)	
One-day introductory course only	5 (15.0)	
Web-based SATS course only	3 (9.0)	
Other combinations	10 (29.0)	
Years as registered nurse	12.44 ± 7.58	2–38
Years in EMS	9.65 ± 8.74	1–40
Experience with other triage systems	20 (58.8)	RETTS*, Other
Years using SATS	5.35 ± 2.19	1–7

*Rapid Emergency Triage and Treatment System

**Fig. 2:**
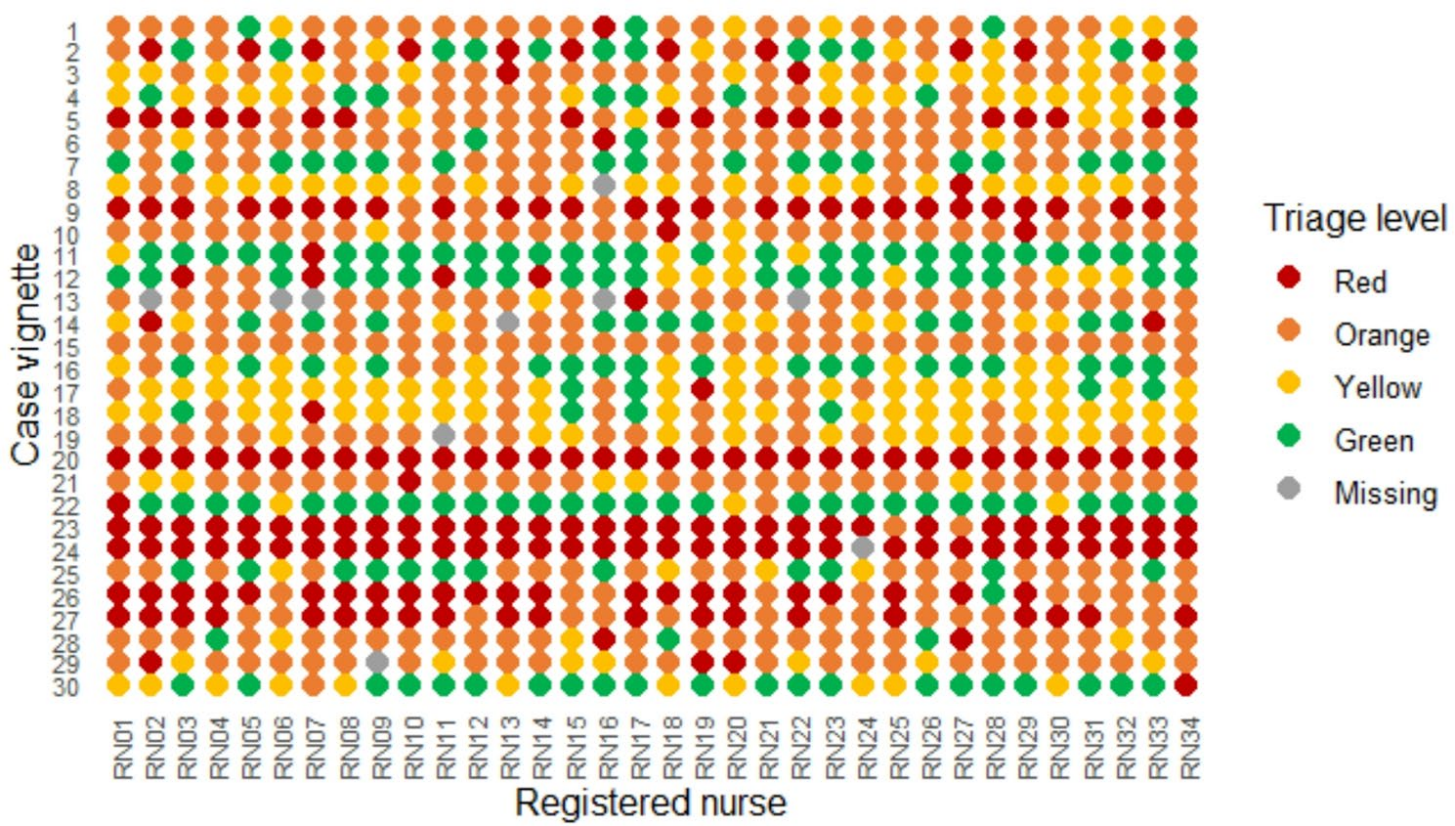
Total triage color assigned by 34 registered nurses for 30 case vignettes, including instances of missing classifications

Together, they completed 1,020 triage assessments based on the 30 case vignettes. Ten assessments were excluded because of ambiguous or invalid responses (Fig. 2).

### Agreement between raters in triage assessments

Krippendorff´s α for total triage with uptriage was 0.602 (95% CI [0.423, 0.712], bootstrap, 1000 resamples). This suggests poor inter-rater agreement. When evaluating triage reliability excluding manual uptriage, but triage based solely on TEWS and potential discriminator, Krippendorff´s α was 0.621 (95% CI [0.442, 0.755], bootstrap, 1000 resamples). When triage levels were dichotomized into high- and low-acuity (orange/red triage vs. green/yellow triage), inter-rater reliability decreased to a Krippendorff´s α of 0.47 (95% [CI 0.33–0.60] bootstrap, 1000 resamples).

**Fig. 3:**
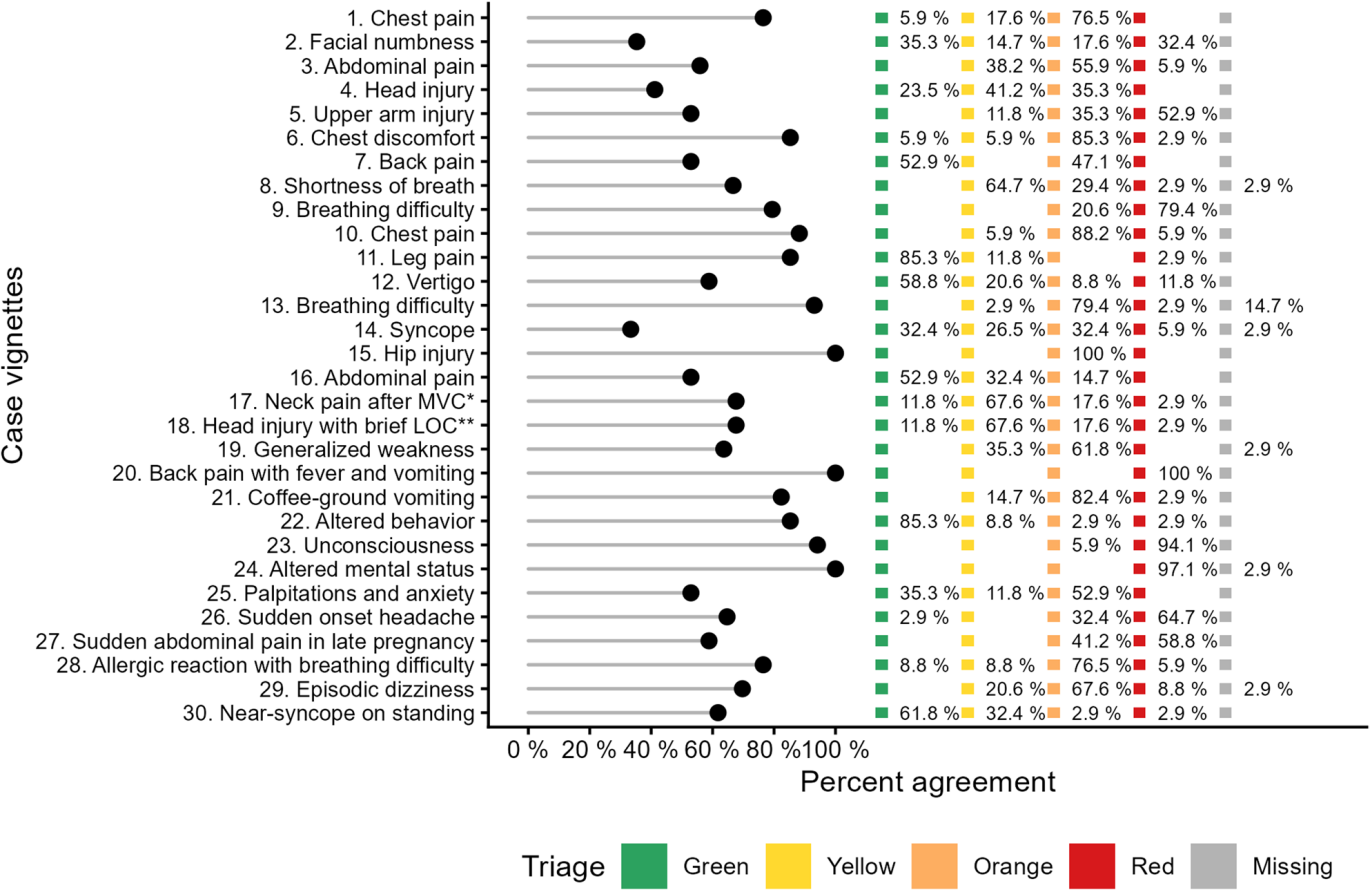
Percent agreement and triage distribution for each of the thirty case vignettes, descriptive vignette labels are provided alongside each case. *Motor vehicle collision (MVC), **Loss of consciousness (LOC)

Nine vignettes were assigned to all four triage levels (vignettes 2, 6, 12, 14, 17, 18, 22, 28, and 30), whereas 13 vignettes were assigned across three triage levels (vignettes 1, 3, 4, 5, 8, 10, 11, 13, 16, 21, 25, 26, and 29).The percentage agreement varied substantially between the vignettes (Fig. 3). Full agreement (100%) was observed in three patient cases (vignettes 15, 20, and 24). Near-complete agreement was noted in two vignettes (13 and 23). In contrast, three vignettes demonstrated low agreement, with less than 50% concordance (vignettes 2, 4, and 14). The detailed percentage agreement for all the cases is provided in Additional file 4.

### Differences in triage using trauma and mobility

Trauma was marked in a limited number of vignettes, but when present, it was often applied by the majority of the raters (vignettes 4, 5, 15, 17, and 18, each 100%) (Table 2).

Mobility assessments demonstrated variation between vignettes. Some presentations were almost uniformly classified as “Independent” (vignettes 1, 10, 16, 20, 22, and 25), whereas others were predominantly rated as “Stretcher/Immobile” (vignettes 13, 15, 23, and 24). Several vignettes showed a wide distribution across all three mobility categories, indicating substantial rater-dependent interpretation (vignettes 8, 17, 21 and 26) (Table 2).

**Table 2:** Proportion of manual uptriage, assessment of trauma, mobility and missing values (*n* = 34)

Vignette	Manual uptriage % (*n*)	Trauma = Yes %	Mobility: Independent %	Mobility: With help %	Mobility: Bedbound/spinal immobilization %
1.	23,5 (8)	-	100	-	-
2.	38,2 (13)	-	94	3	3
3.	17,6 (6)	-	94*	6	-
4.	67,6 (23)	100	94	6	-
5.	17,6 (6)	100	82*	12	6
6.	8,8 (3)	-	38	62	-
7.	-	-	29	68	3
8.◊	20,6 (7)	-	18	71	12
9.	-	18*	-	26	74
10.	11,8 (4)	-	100	-	-
11.	5,9 (2)	-	59	35	6
12.	32,4 (11)	-	3	88	9
13.**	5,9 (2)	-	6	9	85
14. ◊	47,1 (16)	56	91*	6	3
15.	-	100	-	9	91
16.	35,3 (12)	-	97	3	-
17.	8,8 (3)	100	21	9	71
18.	20,6 (7)	100	38	3	59
19. ◊	-	9	6	35	59
20.	-	-	97	3	-
21.	14,7 (5)	-	9*	32	59
22.	11,7 (4)	-	97	-	3
23.	-	68	3	-	97
24. ◊	5,9 (2)	-	3	3	94
25.	11,8 (4)	-	100*	-	-
26.	58,8 (20)	-	53	35	12
27.	8,8 (3)	-	88	12	-
28.	14,7 (5)	-	97	3	-
29. ◊	73,5 (25)	-	18	82	-
30.	23,5 (8)	-	91	9	-

*One rating missing

◊ One rating missing in total triage

**Five ratings missing in total triage

Variation in mobility assessment resulted in differences in final triage levels across the high versus low acuity threshold in four vignettes. In three of these cases (vignettes 8, 13, and 23), the discrepancy was driven by a small number of outlying assessments, with the vast majority of raters assigning the same acuity level.

**Fig. 4:**
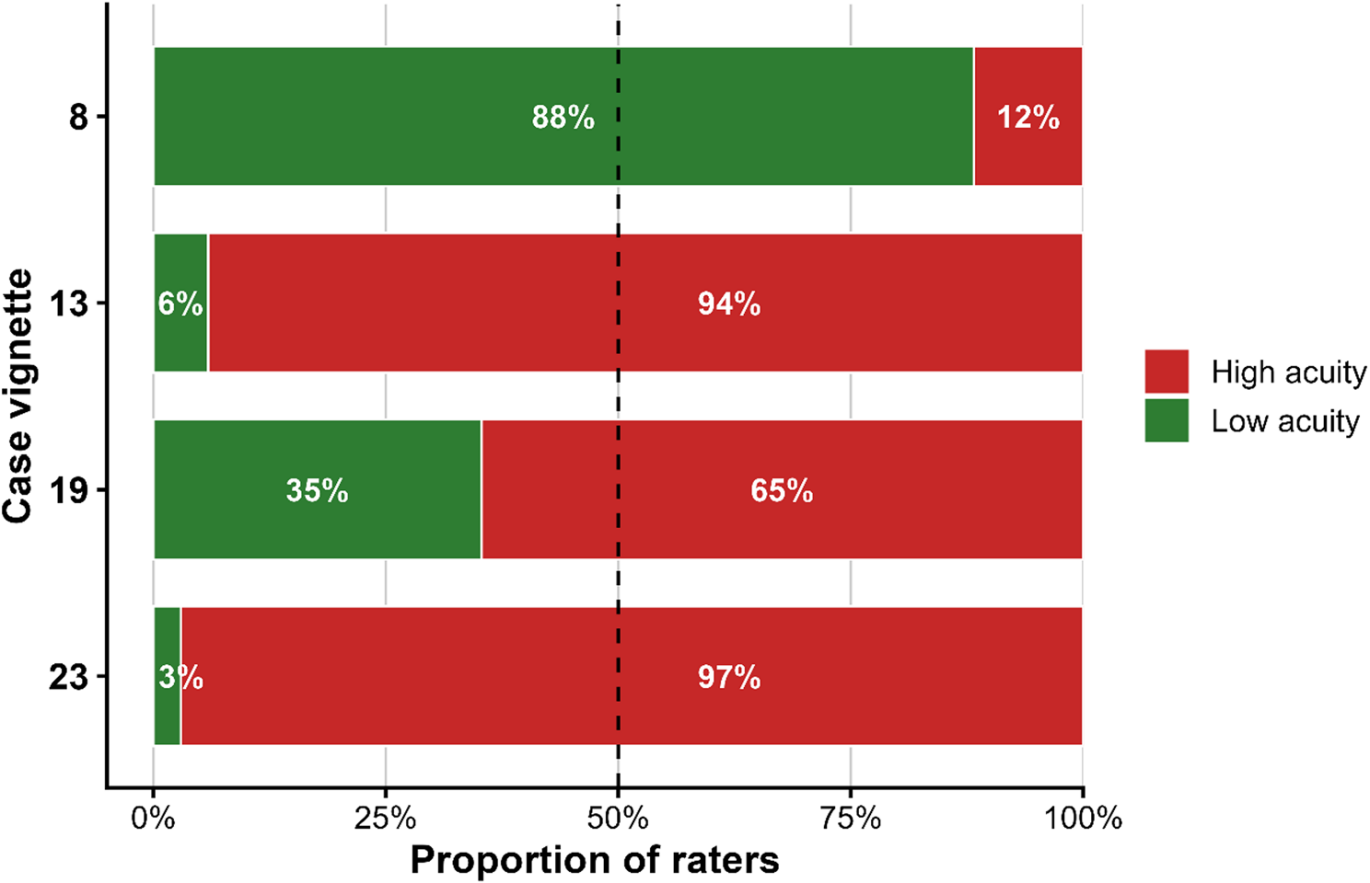
Proportion of high- and low-acuity ratings after adding mobility assessment to baseline TEWS, affecting urgent vs. non-urgent classification in vignettes 8, 13, 19, and 23

In contrast, one vignette (vignette 19) demonstrated a more substantial level of disagreement, with a more uniform distribution of high and low acuity classifications among raters (Fig. 4).

### Differences in triage using manual uptriage

Across the thirty case vignettes, manual up-triage occurred in a varying proportion of assessments, ranging from 0% to 72.7%. Several vignettes, including vignette 4 (67.6%), vignette 14 (47%), vignette 16 (35.3%), vignette 26 (58.8%), and vignette 29 (72.7%), frequently deviated from the triage algorithm (Table 2). These vignettes demonstrated a total triage assessment across 3 triage levels or more.

Manual up-triage occurred in 199 of 1010 assessments (19.7%). Most manual uptriage decisions occurred among cases initially classified as green triage level (68.8% of all uptriage decisions), followed by yellow (21.1%) and orange (10.1%). The majority of uptriage decisions involved escalation by one triage level (green to yellow, yellow to orange, orange to red) (Table 3).

**Table 3:**
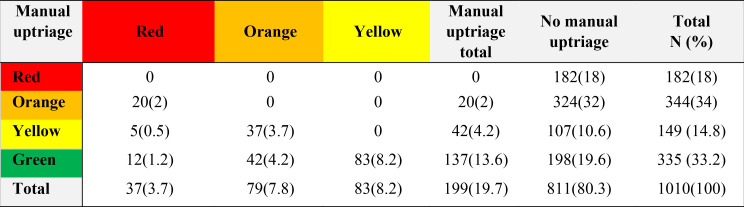
Crosstabulation showing the number (N) and percentage (%) of manual uptriage from each triage category and its distribution across higher triage levels

### Differences in triage when assigning a discriminator

Four vignettes (vignette 2, 7, 14 and 25) demonstrated a near-equal distribution of assessments across the low-acuity and high-acuity threshold (Fig. 3). Further analysis of the triage process of these vignettes suggested that variability emerged primarily following discriminator assignment, resulting in a divergence between low- and high-acuity classifications. Manual uptriage also contributed to variation in final triage level (Table 4).

**Table 4:** Complete SATS triage process for vignettes in which final triage allocation crossed the boundary between high-acuity (orange/red) and low-acuity (yellow/green) triage levels. The table illustrates how TEWS-based triage, discriminator assessment, and manual up-triage contributed to variation in the final triage decision

The triage process in vignettes with high dispersion across low- and high-acuity triage levels (*n* = 34)					
Vignette	Triage level by TEWS (*n*)	No. of discriminators used (*n*)	Triage level by discriminator* (*n*)	Triage level by manual up-triage (*n*)	Final triage after up-triage (*n*)
2.	Green (33) Yellow (1)	1	Green (25) Red (9)	Yellow (5) Orange (6) Red (2)	Green (12) Yellow (5) Orange (6) Red (11)
7.	Green (34)	1	Green (18) Orange (16)		Green (18) Orange (16)
14.	Green (32)◊ Yellow (1)	2	Green (28)◊ Orange (3) Red (2)	Yellow (8) Orange (8)	Green (11)◊ Yellow (9) Orange (11) Red (2)
25.	Green (34)	1	Green (17) Orange (17)	Yellow (5)	Green (12) Yellow (5) Orange (17)

*Green triage in this category implies that no discriminator was used (See supplementary File 1)

◊One triage assessment missing

### Association between RN characteristics and triage outcomes

A mixed-effects logistic regression model was used to examine the association between RN characteristics (years of experience in EMS, education level, and gender) and the likelihood of assigning a high-acuity triage level (high vs. low). The model included random intercepts for both RNs and case vignettes to account for the cross-classified structure of the data (Table 5).

The results revealed no statistically significant association between RN characteristics and assigned triage level. Years of experience was not associated with the likelihood of assigning a high-acuity triage level (OR = 1.01, 95% CI 0.98–1.05, *p* = 0.426). Similarly, no significant differences were observed between education levels (e.g., ambulance specialist nurse vs. registered nurse: OR = 1.24, 95% CI 0.65–2.36, *p* = 0.506), and gender was not associated with a specific triage level (OR = 1.03, 95% CI 0.58–1.82, *p* = 0.917).

**Table 5:** Mixed-effects logistic regression analysis of factors associated with high triage level

Variable	OR	95% CI	*p*-value
**Years in EMS**	1.01	0.98–1.05	0.426
**Education level**			
RN (reference)	1.00	–	–
Ambulance specialist nurse	1.24	0.65–2.36	0.506
Anesthesia specialist nurse	0.47	0.16–1.34	0.157
Emergency specialist nurse	0.39	0.09–1.66	0.204
Other specialist nurse	1.99	0.68–5.79	0.209
**Gender**			
Male (reference)	1.00	–	–
Female	1.03	0.58–1.82	0.917

**Random effects**: Variance (RN) = 0.25; Variance (Case vignette) = 7.84

**Model: **Mixed-effects logistic regression with random intercepts for nurses and case vignettes

## Discussion

This study examined the inter-rater reliability of a locally modified version of the South African Triage Scale (SATS) when it was applied in a prehospital EMS context. Overall, the findings indicate poor agreement between raters, with substantial variability across individual case vignettes. There was no observed association between gender of RNs, years of clinical experience, educational level and triage assignment.

### Inter-rater reliability of SATS in the prehospital setting

A Krippendorff’s α of 0.60 indicates poor agreement when triage levels are treated as ordinal data. Although manual uptriage contributed to variation in total triage levels, Krippendorff´s α increased only marginally (α 0.62) after exclusion of this factor. When triage levels were dichotomized into high- and low-acuity categories using a nominal scale, inter-rater reliability decreased to a Krippendorff´s α of 0.47.

Krippendorff’s cutoff values are more conservative than the corresponding kappa thresholds [40]. Still, other reliability studies of triage systems using Krippendorff´s α reported higher reliability estimates, ranging from 0.68 to 0.78 [41–44]. Although direct comparisons between triage systems may be difficult due to differences in algorithms, clinical settings, and educational level among users, the overall aim of triage systems remains the same: to distinguish acute patients requiring urgent care from non-acute patients with non-urgent conditions. When dichotomizing the triage levels into high- and low acuity categories, inter-rater reliability decreased, highlighting disagreement on whether some patients should be categorized as requiring urgent care or not. The reliability observed in the present study indicates room for improvement. Overall, the results suggest that the agreement between RNs was limited and insufficient to be considered robust according to commonly accepted thresholds for reliability.

Previous studies evaluating prehospital triage systems have reported lower reliability estimates than triage decisions conducted in ED settings [22, 24, 25].

The prehospital environment is characterized by time pressure, limited access to advanced diagnostic resources, and contextual uncertainty—factors that are likely to contribute to increased variability in triage decisions in real-world practice. However, these conditions were not present in the current study, which was based on fictional case vignettes. Furthermore, the present study evaluated a modified version of the SATS, which limits direct comparisons with studies that have assessed the original version of the instrument.

### Case-dependent variability

The percentage agreement varied substantially across the thirty case vignettes, indicating that reliability was highly dependent on the characteristics of the individual cases. Higher levels of agreement were generally observed in vignettes describing clear physiological abnormalities or well-defined high-acuity conditions. In contrast, vignettes involving non-specific symptoms, near-normal vital signs or less clearly defined presentations relied more heavily on clinical interpretation, discriminator assessment, and individual judgement, which may have contributed to the lower agreement observed in these vignettes. Similar findings have been reported in studies of other triage systems [45, 46], where reliability decreases in patient scenarios lacking clear discriminators or objective thresholds, which also highlights the difficulties in creating a gold-standard triage system. From a clinical perspective, this variability is relevant, as it implies that patients with comparable presentations may receive different triage levels depending on the assessor.

Paper-based case vignettes have previously been shown to be a comparable and acceptable method for reliability studies [47]. Nevertheless, this approach limits the possibility of obtaining additional patient information through follow-up questions, which might have influenced the assigned triage level. Importantly, the present study was designed to evaluate the reliability of SATS rather than to determine the presence of under- or overtriage. All participants were provided with identical information and had the same conditions under which to make their triage assessments. The case vignettes were designed to reflect real-world clinical practice in Sweden, where a large proportion of ambulance patients receive green or yellow triage levels [37].

### Judgment-based components and manual up-triage

The inclusion of judgment-based parameters such as mobility and trauma aims to increase sensitivity and reduce the risk of undertriage [26]. However, these components introduce a degree of subjectivity. In the present study, assessments of mobility demonstrated substantial variability, particularly in cases involving weakness, dizziness, or reduced functional capacity without evident injury. This might partly be explained by time constraints or insufficient thorough review of the vignettes by some respondents. However, the degree of variability also suggests genuine differences in interpretation of clinical presentations. The use of paper vignettes may also have contributed to this variability, as mobility assessment may be more accurately evaluated in real-life patient encounters. Nevertheless, this category of patients has previously been shown to be challenging to assess [48]. The trauma parameter was more uniformly applied across raters. In the few vignettes where it was used inconsistently, it did not influence the final triage level. Similarly, although variability in mobility assessment occasionally affected the calculated TEWS-score, it was not the primary contributor to the differences between low- and high-acuity triage allocation.

The increased use of manual up-triage observed in several vignettes may reflect a compensatory response by clinicians when standardized triage parameters fail to adequately capture perceived patient risk. This phenomenon has previously been described among patients with non-specific presentations and normal vital signs [49]. Manual up-triage occurred frequently in selected scenarios, indicating a pronounced reliance on clinical judgment beyond algorithm-based scoring. The vignettes were designed to capture the patients’ overall clinical status by presenting information relevant to assessment that is not necessarily reflected in vital signs, such as frailty, underlying comorbidities, and current medication use. Such information plays an important role in clinical judgment and may have contributed to the frequency of manual up-triage decisions.

Variability in triage decisions is clinically relevant, as triage errors have been associated with adverse patient outcomes [50], including longer ED length of stay [51], which may in turn increase staff workload and increase the risk of delayed care. Judgment-based triage components may therefore represent important contributors to patient safety risks in prehospital care. Although such flexibility may be necessary in prehospital care, it appears to be a key contributing factor to the reduced inter-rater reliability observed. These findings indicate that while clinical flexibility is essential in the prehospital setting, it may simultaneously contribute to variation in triage assessments, thereby challenging standardization.

### Experience, education, and triage decisions

No associations were detected between gender, years of experience in the EMS or specialist education and the assigned triage level. This suggests that variation in triage decisions cannot be explained primarily by the individual background characteristics in this study. Similar results have been reported in a reliability study of the Australian Triage Scale [52]. However, other studies have shown conflicting results regarding the relationship between clinical experience and triage accuracy [53, 54]. One possible explanation for the findings of the present study is that more experienced clinicians may rely more heavily on individual clinical decision-making processes, which can vary between assessors, whereas formal education alone appears insufficient to standardize judgment-based decisions, as previously described by Considine et al. [40]. Individual factors such as gender and personality traits may also contribute to variability in clinical decision-making [55]. However, as only ten male participants were included in the present study, no conclusions can be drawn regarding the potential influence of gender on assigned triage levels. Prior work experience before employment in the EMS was not reported in the study, although the type of previous professional experience may influence clinical decision-making. Although all RNs had received some form of SATS training, the type and extent of training varied. This variation may have influenced how the triage system was interpreted and applied, potentially contributing to differences in triage decisions. Similar variability in triage decisions has previously been described, and in line with the present findings, the authors of the study questioned whether clinical experience alone improves triage consistency [46].

### Clinical implications and future research

These findings raise important considerations regarding the implementation of locally adapted triage systems in prehospital care. While local adaptation may increase contextual relevance, such modifications should be accompanied by systematic evaluations of reliability and validity, such as in this study. Limited inter-rater reliability may affect patient prioritization, allocation of resources, and subsequent care processes. Incorrect triage assignments may also compromise patient safety. Undertriage should be minimized, as it may delay necessary care for critically ill patients, whereas a high degree of overtriage may unnecessarily strain available resources and reduce system efficiency. In addition, frequent manual uptriage due to uncertainty may have important clinical implications, suggesting that the triage algorithm should not rely heavily on subjective adjustment to identify high-acuity patients. These findings of this study may inform future refinements of the current version of SATS, with the aim of improving consistency and supporting safer triage decisions.

Future research should investigate whether this modified version of SATS is associated with a high degree of under- or overtriage in the prehospital setting, triage accuracy and whether it matches the triage decisions made in the ED. In addition, in-depth qualitative studies exploring ambulance nurses’ experiences using SATS may contribute to clearer definitions or refinements of judgment-based parameters, thereby potentially improving reliability. Additional assessment using real-life clinical cases might be necessary, as the reliability estimates obtained in this study did not meet established acceptance criteria [47].

### Strengths and limitations

The strengths of this study include adherence to the GRAAS reporting guidelines and the inclusion of a wide range of detailed prehospital case vignettes together with 1,010 triage assessments. However, there are limitations to this study, including the use of fictional vignette cases, which may not fully reflect the clinical complexity of real-life situations. In real-life clinical practice, RNs may consult colleagues or seek physician support when clinical presentations are unclear or uncertain. The level of detail provided in the case vignettes may have contributed to variability in triage decisions where less complex or, more clearly, high-acuity scenarios might have resulted in greater agreement. Although participants were able to save their responses and complete the questionnaire in multiple sessions, the overall time required to complete the questionnaire (approximately 70 min) may have contributed to respondent fatigue, potentially affecting the consistency of the responses. Together with the limited number of participants, this may affect the generalizability of the findings. Although the distribution of men and women reflected the demographics of the studied region and participants represented a wide range of clinical experience, a larger sample size might have allowed for a more detailed analysis of potential associations between triage performance and clinical experience, educational level, sex, and prior experience with triage. Furthermore, the modification of SATS limits comparability with studies using other versions of the system, and the use of Krippendorff´s alpha for statistical analysis further reduces comparability, as this measure is uncommon in prehospital triage research.

## Conclusion

In this prehospital inter-rater reliability study, a locally modified version of SATS demonstrated moderate to low agreement among registered nurses. Agreement varied considerably across patient cases, particularly in patient cases presenting near-normal vital signs. Neither clinical experience nor specialist education was associated with triage patterns. These findings underscore the need for careful implementation and continuous evaluation of modified triage systems in prehospital emergency care.

## Supplementary Information

Below is the link to the electronic supplementary material.

Supplementary Material 1

Supplementary Material 2

Supplementary Material 3

Supplementary Material 4

## Data Availability

The datasets used and analyzed during the current study are available from the corresponding author upon reasonable request.

## Abbreviations

EMD: Emergency medical dispatch
EMS: Emergency medical services
ED: Emergency department
SATS: South African Triage Scale
RN: Registered nurse
GRAAS: Guidelines for Reporting Reliability and Agreement Studies
ECG: Electrocardiogram
TEWS: Triage early warning score
RR: Respiratory rate
BP: Blood pressure
Temp: Temperature
α: Alpha
RETTS: Rapid Emergency Triage and Treatment System
SD: Standard deviation
MVC: Motor vehicle collision
LOC: Loss of consciousness
CI: Confidence interval
OR: Odds ratio

## References

[1] Robertson-Steel I. Evolution of triage systems. Emerg Med J. 2006;23(2):154–5. 10.1136/emj.2005.030270. 16439754 10.1136/emj.2005.030270 PMC2564046

[2] Kim K, Oh B. Prehospital triage in emergency medical services system: A scoping review. Int Emerg Nurs. 2023;69:101293. 10.1016/j.ienj.2023.101293. PubMed PMID: 37150145. 37150145 10.1016/j.ienj.2023.101293

[3] Bhaumik S, Hannun M, Dymond C, DeSanto K, Barrett W, Wallis LA, et al. Prehospital triage tools across the world: a scoping review of the published literature. Scand J Trauma Resusc Emerg Med. 2022;30(1):32. 10.1186/s13049-022-01019-z. PubMed PMID: 35477474; PubMed Central PMCID: PMC9044621. 35477474 10.1186/s13049-022-01019-z PMC9044621

[4] Lidal IB, Holte HH, Vist GE. Triage systems for pre-hospital emergency medical services - a systematic review. Scand J Trauma Resusc Emerg Med. 2013;21(1):28. 10.1186/1757-7241-21-28. 23587133 10.1186/1757-7241-21-28 PMC3641954

[5] Kingswell CJ, Calleja P, Sahay A. The Impact of Emergency Triage Practices on Patient Safety: A Scoping Review Protocol. J Emerg Nurs. 2025;51(3):498–503. 10.1016/j.jen.2024.12.002. 39955670 10.1016/j.jen.2024.12.002

[6] Bohm K, Kurland L. The accuracy of medical dispatch - a systematic review. Scand J Trauma Resusc Emerg Med. 2018;26(1):94. 10.1186/s13049-018-0528-8. 30413213 10.1186/s13049-018-0528-8 PMC6230269

[7] Odberg KR, Aase K, Grusd E, Vifladt A. The work system of prehospital medication administration: a qualitative mixed methods study with ambulance professionals. BMC Emerg Med. 2025;25(1):54. 10.1186/s12873-025-01213-z. 40188015 10.1186/s12873-025-01213-z PMC11972525

[8] Hichisson A, Wilcock G, Eaton G, Taylor LJ, Jolly JK. Paramedic practice in low light conditions: a scoping review. J Paramed Pract. 2023;15(1):6–15. 10.12968/jpar.2023.15.1.6.

[9] Lumley HA, Flynn D, Shaw L, McClelland G, Ford GA, White PM, et al. A scoping review of pre-hospital technology to assist ambulance personnel with patient diagnosis or stratification during the emergency assessment of suspected stroke. BMC Emerg Med. 2020;20(1):30. 10.1186/s12873-020-00323-0. PubMed PMID: 32336270; PubMed Central PMCID: PMC7183583. 32336270 10.1186/s12873-020-00323-0 PMC7183583

[10] Quadflieg LTM, Beckers SK, Bergrath S, Brockert AK, Schröder H, Sommer A, et al. Comparing the diagnostic concordance of tele-EMS and on-site-EMS physicians in emergency medical services: a retrospective cohort study. Sci Rep. 2020;10(1):17982. 10.1038/s41598-020-75149-8. PubMed PMID: 33093557; PubMed Central PMCID: PMC7581718. 33093557 10.1038/s41598-020-75149-8 PMC7581718

[11] Glawing C, Karlsson I, Kylin C, Nilsson J. Work-related stress, stress reactions and coping strategies in ambulance nurses: A qualitative interview study. J Adv Nurs. 2024;80(2):538–49. 10.1111/jan.15819. 37530409 10.1111/jan.15819

[12] Ivic R, Kurland L, Vicente V, Castrén M, Bohm K. Serious conditions among patients with non-specific chief complaints in the pre-hospital setting: a retrospective cohort study. Scand J Trauma Resusc Emerg Med. 2020;28(1):74. 10.1186/s13049-020-00767-0. PubMed PMID: 32727586; PubMed Central PMCID: PMC7391698. 32727586 10.1186/s13049-020-00767-0 PMC7391698

[13] Boulton AJ, Peel D, Rahman U, Cole E. Evaluation of elderly specific pre-hospital trauma triage criteria: a systematic review. Scand J Trauma Resusc Emerg Med. 2021;29(1):127. 10.1186/s13049-021-00940-z. PubMed PMID: 34461976; PubMed Central PMCID: PMC8404299. 34461976 10.1186/s13049-021-00940-z PMC8404299

[14] Khan IA, Karim HMR, Panda CK, Ahmed G, Nayak S. Atypical Presentations of Myocardial Infarction: A Systematic Review of Case Reports. Cureus. 2023;15(2):e35492. 10.7759/cureus.35492. PubMed PMID: 36999116; PubMed Central PMCID: PMC10048062. 36999116 10.7759/cureus.35492 PMC10048062

[15] Magnusson C, Herlitz J, Axelsson C. Patient characteristics, triage utilisation, level of care, and outcomes in an unselected adult patient population seen by the emergency medical services: a prospective observational study. BMC Emerg Med. 2020;20(1):7. 10.1186/s12873-020-0302-x. 32000684 10.1186/s12873-020-0302-x PMC6993445

[16] Lane D, Ichelson RI, Drennan IR, Scales DC. Prehospital management and identification of sepsis by emergency medical services: a systematic review. Emerg Med J EMJ. 2016;33(6):408–13. 10.1136/emermed-2015-205261. PubMed PMID: 26864327. 26864327 10.1136/emermed-2015-205261

[17] van Veen M, Moll HA. Reliability and validity of triage systems in paediatric emergency care. Scand J Trauma Resusc Emerg Med. 2009;17:38. 10.1186/1757-7241-17-38. PubMed PMID: 19712467; PubMed Central PMCID: PMC2747834. 19712467 10.1186/1757-7241-17-38 PMC2747834

[18] Wireklint SC, Elmqvist C, Parenti N, Göransson KE. A descriptive study of registered nurses’ application of the triage scale RETTS©; a Swedish reliability study. Int Emerg Nurs. 2018;38:21–8. 10.1016/j.ienj.2017.12.003. 29326039 10.1016/j.ienj.2017.12.003

[19] Dalwai MK, Twomey M, Maikere J, Said S, Wakeel M, Jemmy JP, et al. Reliability and accuracy of the South African Triage Scale when used by nurses in the emergency department of Timergara Hospital, Pakistan. South Afr Med J Suid-Afr Tydskr Vir Geneeskd. 2014;104(5):372–5. 10.7196/samj.7604. PubMed PMID: 25212207. 10.7196/samj.7604 25212207

[20] Dalwai M, Tayler-Smith K, Twomey M, Nasim M, Popal AQ, Haqdost WH, et al. Inter-rater and intrarater reliability of the South African Triage Scale in low-resource settings of Haiti and Afghanistan. Emerg Med J. 2018;35(6):379–83. 10.1136/emermed-2017-207062. PubMed PMID: 29549171. 29549171 10.1136/emermed-2017-207062 PMC5969337

[21] Ebrahimi M, Heydari A, Mazlom R, Mirhaghi A. The reliability of the Australasian Triage Scale: a meta-analysis. World J Emerg Med. 2015;6(2):94–9. 10.5847/wjem.j.1920-8642. 2015.02.002 PubMed PMID: 26056538; PubMed Central PMCID: PMC4458479. 26056538 10.5847/wjem.j.1920-8642.2015.02.002 PMC4458479

[22] Buschhorn HM, Strout TD, Sholl JM, Baumann MR. Emergency medical services triage using the emergency severity index: is it reliable and valid? J Emerg Nurs. 2013;39(5):e55–63. 10.1016/j.jen.2011.11.003. PubMed PMID: 22244546. 22244546 10.1016/j.jen.2011.11.003

[23] Grosgurin O, Gayet-Ageron A, Suppan L, Simon J, Villar A, Trombert V, et al. Reliability and performance of the Swiss Emergency Triage Scale used by paramedics. Eur J Emerg Med Off J Eur Soc Emerg Med. 2019;26(3):188–93. 10.1097/MEJ.0000000000000530. PubMed PMID: 29252610; PubMed Central PMCID: PMC6504125. 10.1097/MEJ.0000000000000530 PMC6504125 29252610

[24] Leeies M, Ffrench C, Strome T, Weldon E, Bullard M, Grierson R. Prehospital Application of the Canadian Triage and Acuity Scale by Emergency Medical Services. CJEM. 2017;19(1):26–31. 10.1017/cem.2016.345. PubMed PMID: 27508353. 27508353 10.1017/cem.2016.345

[25] Mould-Millman NK, Dixon JM, Burkholder T, Pigoga JL, Lee M, de Vries S, et al. Validity and reliability of the South African Triage Scale in prehospital providers. BMC Emerg Med. 2021;21(1):8. 10.1186/s12873-021-00406-6. PubMed PMID: 33451294; PubMed Central PMCID: PMC7811258. 33451294 10.1186/s12873-021-00406-6 PMC7811258

[26] Gottschalk SB, Wood D, DeVries S, Wallis LA, Bruijns S, Cape Triage Group. The Cape Triage Score: a new triage system South Africa. Proposal from the Cape Triage Group. Emerg Med J EMJ. 2006;23(2):149–53. 10.1136/emj.2005.028332. PubMed PMID: 16439753; PubMed Central PMCID: PMC2564045. 16439753 10.1136/emj.2005.028332 PMC2564045

[27] Massaut J, Valles P, Ghismonde A, Jacques CJ, Louis LP, Zakir A, et al. The modified south African triage scale system for mortality prediction in resource-constrained emergency surgical centers: a retrospective cohort study. BMC Health Serv Res. 2017;17(1):594. 10.1186/s12913-017-2541-4. 28835247 10.1186/s12913-017-2541-4 PMC5569494

[28] EMSSA. Emergency medicin of South Africa. The South African triage scale (SATS). [Internet]. 2017. Available from: https://emssa.org.za/special-interest-groups/the-south-african-triage-scale-sats/.

[29] Habbouche S, Carlson T, Johansson D, Kjaerbeck S, Malm M, Svensson PA, et al. Comparison of the novel WEst coast System for Triage (WEST) with Rapid Emergency Triage and Treatment System (RETTS©): an observational pilot study. Int J Emerg Med. 2022;15(1):47. 10.1186/s12245-022-00452-2. PubMed PMID: 36096726; PubMed Central PMCID: PMC9465908. 36096726 10.1186/s12245-022-00452-2 PMC9465908

[30] Ohlin E. Region Gavleborg byter triageverktyg. Läkartidningen. 2018;115.

[31] SATS Norge [Internet]. [cited 2026 Jan 14]. Available from: https://www.helse-bergen.no/4a4642/siteassets/seksjon/mottaksklinikken/documents/2020.09.01-sats-norge-versjon-4.0-_-manual.pdf.pdf.

[32] Twomey M, Wallis LA, Thompson ML, Myers JE. The South African Triage Scale (adult version) provides reliable acuity ratings. Int Emerg Nurs. 2012;20(3):142–50. 10.1016/j.ienj.2011. 08.002 PubMed PMID: 22726946. 22726946 10.1016/j.ienj.2011.08.002

[33] Markussen DL, Brevik HS, Bjørneklett RO, Engan M. Validation of a modified South African triage scale in a high-resource setting: a retrospective cohort study. Scand J Trauma Resusc Emerg Med. 2023;31(1):13. 10.1186/s13049-023-01076-y. 36941710 10.1186/s13049-023-01076-y PMC10026449

[34] Kottner J, Audigé L, Brorson S, Donner A, Gajewski BJ, Hróbjartsson A, et al. Guidelines for Reporting Reliability and Agreement Studies (GRRAS) were proposed. J Clin Epidemiol. 2011;64(1):96–106. 10.1016/j.jclinepi.2010.03.002. PubMed PMID: 21130355. 21130355 10.1016/j.jclinepi.2010.03.002

[35] Statistikdatabasen [Internet]. [cited 2025 Dec 19]. Life expectancy at birth by region and sex 1998-2002-2020–2024. Available from: https://www.statistikdatabasen.scb.se:443/pxweb/en/ssd/START__BE__BE0101__BE0101I/Medellivsl/.

[36] Lindström V, Bohm K, Kurland L. Prehospital care in Sweden: From a transport organization to advanced healthcare. Notf Rettungsmedizin. 2015;18(2):107–9. 10.1007/s10049-015-1989-1.

[37] Ambureg. Årsrapport för Ambureg 2022 [Internet]. 2023. Available from: https://rcsyd.se/ambureg/wp-content/uploads/sites/16/2024/02/Arsrapport-for-Ambureg-2022.pdf.

[38] Krippendorff K. Content analysis: an introduction to its methodology [Internet]. 2455 Teller Road, Thousand Oaks California 91320: SAGE Publications, Inc.; 2019 [cited 2025 Dec 29]. Available from: https://methods.sagepub.com/error/handleStatusCode?code=40410.4135/9781071878781.

[39] Marzi G, Balzano M, Marchiori D. K-Alpha Calculator–Krippendorff’s Alpha Calculator: A user-friendly tool for computing Krippendorff’s Alpha inter-rater reliability coefficient. MethodsX. 2024;12:102545. 10.1016/j.mex.2023.102545. 39669968 10.1016/j.mex.2023.102545 PMC11636850

[40] Hallgren KA. Computing Inter-Rater Reliability for Observational Data: An Overview and Tutorial. Tutor Quant Methods Psychol. 2012;8(1):23–34. 10.20982/tqmp.08.1.p023. PubMed PMID: 22833776; PubMed Central PMCID: PMC3402032. 22833776 10.20982/tqmp.08.1.p023 PMC3402032

[41] Habib H, Prabowo Y, Sulistio S, Albar IA, Mulyana RM, Nurlaelah S, et al. Accuracy of nurse-based Cipto Triage Method in the emergency department. F1000Research. 2024;12:328. 10.12688/f1000research.130992.2.

[42] Jordi K, Grossmann F, Gaddis GM, Cignacco E, Denhaerynck K, Schwendimann R, et al. Nurses’ accuracy and self-perceived ability using the Emergency Severity Index triage tool: a cross-sectional study in four Swiss hospitals. Scand J Trauma Resusc Emerg Med. 2015;23(1):62. 10.1186/s13049-015-0142-y. 26310569 10.1186/s13049-015-0142-y PMC4551516

[43] Mistry B, Hinson J, Balhara K, De Stewart SA, Anton X, Levin S, et al. 165 Assessing Accuracy and Inter-rater Reliability of the Emergency Severity Index in Triage in the Al-Rahba Emergency Department: A Cross-Sectional Observational Study. Ann Emerg Med. 2016;68(4):S65. 10.1016/j.annemergmed.2016.08.178.

[44] Mistry B, Stewart De Ramirez S, Kelen G, Schmitz PSK, Balhara KS, Levin S, et al. Accuracy and Reliability of Emergency Department Triage Using the Emergency Severity Index: An International Multicenter Assessment. Ann Emerg Med. 2018;71(5):581–e5873. 10.1016/j.annemergmed.2017.09.036. PubMed PMID: 29174836. 29174836 10.1016/j.annemergmed.2017.09.036

[45] Dippenaar E. Reliability and validity of three international triage systems within a private health-care group in the Middle East. Int Emerg Nurs. 2020;51:100870. 10.1016/j.ienj.2020.100870. 32312687 10.1016/j.ienj.2020.100870

[46] Olsson M, Svensson A, Andersson H, Dehre A, Elmqvist C, Rask M, et al. Educational intervention in triage with the Swedish triage scale RETTS©, with focus on specialist nurse students in ambulance and emergency care – A cross-sectional study. Int Emerg Nurs. 2022;63:101194. 10.1016/j.ienj.2022.101194. 35802957 10.1016/j.ienj.2022.101194

[47] Worster A, Sardo A, Eva K, Fernandes CMB, Upadhye S. Triage tool inter-rater reliability: a comparison of live versus paper case scenarios. J Emerg Nurs. 2007;33(4):319–23. 10.1016/j.jen. 2006.12.016 PubMed PMID: 17643791. 17643791 10.1016/j.jen.2006.12.016

[48] Puig-Campmany M, Blázquez-Andión M, Ris-Romeu J. Triage tools: a cautious (and critical) view towards their use in old patients. Eur Geriatr Med. 2022;13(2):319–22. 10.1007/s41999-021-00572-7. 34609734 10.1007/s41999-021-00572-7

[49] Ivic-Morén R, Bohm K, Vicente V, Arvidsson E, Castrén M, Kurland L. Serious conditions among conveyed and non-conveyed patients presenting with nonspecific chief complaints to the ambulance service. BMC Emerg Med. 2024;24(1):199. 10.1186/s12873-024-01106-7. 39443901 10.1186/s12873-024-01106-7 PMC11515605

[50] Khajehgoodari M, Najafi B, Matanagh AS, Lotfi M, Shabanloei R. Nurse triage errors and their relationship with patient outcomes in emergency departments. Int J Afr Nurs Sci. 2025;23:100902. 10.1016/j.ijans.2025.100902.

[51] Ausserhofer D, Zaboli A, Pfeifer N, Solazzo P, Magnarelli G, Marsoner T, et al. Errors in nurse-led triage: An observational study. Int J Nurs Stud. 2021;113:103788. 10.1016/j.ijnurstu.2020.103788. 33120136 10.1016/j.ijnurstu.2020.103788

[52] Ekins K, Morphet J. The accuracy and consistency of rural, remote and outpost triage nurse decision making in one Western Australia Country Health Service Region. Australas Emerg Nurs J. 2015;18(4):227–33. 10.1016/j.aenj.2015.05.002. 26220101 10.1016/j.aenj.2015.05.002

[53] Bednarek-Chałuda M, Żądło A, Antosz N, Clutter P. Polish Perspective: The Influence of National Emergency Severity Index Training on Triage Practitioners’ Knowledge. J Emerg Nurs. 2024;50(3):413–24. 10.1016/j.jen.2023.12.002. 38349291 10.1016/j.jen.2023.12.002

[54] Suamchaiyaphum K, Jones AR, Markaki A. Triage Accuracy of Emergency Nurses: An Evidence-Based Review. J Emerg Nurs. 2024;50(1):44–54. 10.1016/j.jen.2023.10.001. 37930287 10.1016/j.jen.2023.10.001

[55] Blohm M, McGrath A, Mukka S, Jolbäck P. 71273 - Female and male general surgeons differ in personality traits - a Swedish cross-sectional study. Br J Surg. 2024;111(Supplement7):znae175100. 10.1093/bjs/znae175.100.

[56] World Medical Association. World Medical Association Declaration of Helsinki: ethical principles for medical research involving human subjects. JAMA. 2013;310(20):2191–4. 10.1001/jama.2013.281053. PubMed PMID: 24141714. 24141714 10.1001/jama.2013.281053

