# Supplementary material for: An inter-rater reliability study of a modified version of SATS as a prehospital triage tool
Source: Scand J Trauma Resusc Emerg Med. 2026 Jun 12;34:106. doi: 10.1186/s13049-026-01648-8 (PMC13263948; doi:10.1186/s13049-026-01648-8)
Supplement: Supplementary file 1 — Supplementary Material 1 [file 13049_2026_1648_MOESM1_ESM.docx]

**Supplementary information**

**Additional file 1. Additional file 3.**

***Case vignettes***

**Case 1.**

Male, 82 years old

*Situation*

The patient awoke at 03:00 with diffuse chest pain. He attempted to return to sleep but contacted emergency medical services (EMS) later in the morning when the pain persisted despite his regular medication.

*Background*

Medication-treated hypertension and type 2 diabetes mellitus treated with metformin. Current medications include acetylsalicylic acid and enalapril. Slightly overweight. Lives alone in an apartment.

*Current status*

The chest pain began approximately five hours prior to EMS arrival and has remained unchanged. The pain is not exacerbated by movement or deep inspiration and is not reproducible on palpation. The patient describes a dull, aching pain localized to the left side of the chest, occasionally radiating centrally, with a mild stinging sensation in the left hand. It eases at times when he distracts himself but does not disappear.
He reports mild dyspnoea compared with baseline. Pain intensity was assessed using the Numeric Rating Scale (NRS), score 4 to 5 of 10.

**A:** Airway patent; the patient speaks in full sentences without difficulty.

**B:** Clear breath sounds bilaterally. Reports mild dyspnoea.

**C:** Regular radial pulse, normal skin color. Electrocardiogram (ECG) demonstrates sinus rhythm without acute ST-segment changes.

**D:** Alert and oriented. Reaction Level Scale (RLS) score of 1.

**E:** No abnormalities detected.

Following administration of sublingual Nitroglycerin, chest pain decreased to NRS 3/10.

*Vital signs*

Airway: patent
Respiratory rate (RR): 20
SpO₂: 94%
Pulse: 86 beats per minute (bpm)
Blood Pressure (BP): 150/86 mmHg
Blood glucose (BG): 10.2 mmol/L
Temperature (Temp): 37.6 °C
Pain: NRS 3/10

**Case 2.**

Female, 60 years old.

*Situation*

The patient experienced sudden onset of tingling and numbness on the left side of the face while driving home from work. She also experiences reduced hearing in the left ear. She contacted EMS from the side of the road due to feeling unsafe continuing to drive.

*Background*

No known chronic illnesses. No regular medication. History of episodic migraine. Employed full-time and reports work-related stress.

*Current status*

**A:** Airway patent.

**B:** Normal respiratory pattern with clear breath sounds bilaterally.

**C:** Regular radial pulse, normal skin perfusion. ECG demonstrates sinus rhythm.

**D:** Alert and oriented (RLS 1). Facial motor function is intact with reduced sensation on the left side. Normal motor strength in all extremities with preserved grip strength. Speech fluent without dysarthria. No visual disturbances. Markedly reduced hearing in the left ear. The patient is ambulatory but reports mild unsteadiness and dizziness while walking.

**E:** Afebrile, no pain reported.

*Vital signs*

RR: 20
SpO₂: 97%
Pulse: 86 bpm

Heart rate (HR): 86 bpm
BP: 180/75 mmHg
BG: 5.3 mmol/L
Temp: 36.6 °C
Pain: 0

**Case 3.**

Female, 29 years old.

*Situation*

The patient reports lower abdominal pain of approximately 12 hours’ duration. She is found resting in bed upon EMS arrival.

*Background*

Previously healthy.

*Current status*

Gradual onset approximately 12 hours prior to assessment. No relieving factors identified. The patient prefers to lie in a fetal position or to lean forward while sitting. She took two tablets of acetaminophen a few hours earlier, without relief, and reports that the pain has since worsened. She describes a dull, localized abdominal pain in the lower right quadrant. The abdomen is soft but tender to palpation. Bowel sounds are present. No costovertebral angle tenderness. No radiation of pain.

**A*:*** Airway patent.
**B:** Breathing unlabored, clear breath sounds bilaterally.
**C:** Regular radial pulse, normal rate and rhythm.
**D:** Alert and oriented.
**E:** Skin warm and intact; febrile. Appears pale with periorbital dark circles.

The patient denies vaginal bleeding, constipation, or urinary symptoms. She reports generalized weakness and fatigue but is able to stand and ambulate independently.

*Vital signs*

RR: 22
SpO₂: 99%
Pulse: 87 bpm
BP: 110/70 mmHg

Alert
BG: 5.5 mmol/L
Temp: 38.5 °C
Pain: NRS 6/10

**Case 4.**

Male, 84 years old.

*Situation*
The patient’s wife called an ambulance after her husband vomited and reported dizziness following a head injury sustained in a fall. The patient states that approximately four hours prior, he slipped while putting on his socks after showering and struck the back of his head against a wall. He was briefly dazed immediately after the fall but recovered shortly thereafter. Upon ambulance arrival, he was lying in bed and reports that he has been able to ambulate independently around the house.

*Background*
History of hyperlipidemia and benign prostatic hyperplasia. The patient does not take anticoagulant or antiplatelet medications. No use of mobility aids.

*Current status*
The patient experienced mild dizziness immediately after the head injury, with nausea developing several hours later. Since then, he has vomited twice and continues to feel nauseous.

**A:** Airway patent.
**B:** Normal breathing.
**C:** Regular radial pulse. No visible external bleeding. ECG without abnormalities.
**D:** Pupils equal in size and reactive to light. Alert and oriented (RLS 1). A standardized stroke screening tool (Face Arm Speech Test, FAST) was performed. No focal neurological deficits.
**E:** Tenderness to palpation over the occipital region, without palpable hematoma. Denies general headache. No other injuries identified. Denies neck or spinal pain.

*Vital signs*

RR: 16
SpO₂: 97%
Pulse: 86 bpm
BP: 145/70 mmHg
BG: 7.0 mmol/L
Temp: 36.4 °C
Pain: 0

**Case 5**

Male, 49 years old.

*Situation*
The patient fell from a scaffold approximately 1 meter above ground level at his workplace and landed on a piece of rebar. The rebar penetrated the posterior aspect of the upper arm, entering just proximal to the elbow. The distal tip of the rebar is visible beneath the skin. The patient reports no head strike. Upon ambulance arrival, coworkers had cut the rebar approximately 10 cm from the arm, leaving the remaining portion embedded in the limb. The patient is seated on the floor with the injured arm resting on his knee.

*Background*
Previously healthy. No known medical conditions.

*Current status*

**A:** Airway patent; speaking clearly without difficulty.
**B:** Normal breathing, equal breath sounds bilaterally with symmetrical chest expansion.
**C:** Radial pulse full and regular; no visible external bleeding.
**D:** Awake, alert, and oriented. Decreased sensation in the little finger and part of the ring finger; radial pulse palpable in the injured arm. Able to move all fingers. No other neurological deficits. Able to stand and ambulate independently.
**E:** No other visible injuries. Denies pain elsewhere and states that no other body parts were injured in the fall. Skin color normal. Reports pain with movement of the arm as well as with finger movement.

*Vital signs*

RR: 16
SpO₂: 98%
Pulse: 89 bpm
BP: 135/85 mmHg

Alert
Temp: 37.3 °C
Pain: NRS 7/10

**Case 6**

Female, 67 years old.

*Situation*
During the day, the patient has experienced discomfort in the chest and upper back. She reports mild dizziness and nausea and has spent most of the day resting indoors, primarily sitting on a sofa.

*Background*
History of fibromyalgia. Treated hypothyroidism with levothyroxine. Takes antidepressant medication. Lives in a terraced house with a garden. She reports having experienced similar symptoms several years ago, which resolved spontaneously and did not seek medical help. On this occasion, the symptoms are more pronounced.

*Current status*
The discomfort has gradually developed over the past 6–7 hours. The patient reports increased fatigue over the preceding 2–3 days and excessive sleep during this period. She describes generalized weakness and notes that the discomfort worsens with exertion, even with minimal activity such as walking inside the house. She reports mild shortness of breath, though the discomfort does not increase with deep inspiration. The discomfort is diffusely perceived throughout the thorax and is described as an internal chest discomfort with a somewhat pressing sensation centrally, with radiation to the upper back. She also reports increased shoulder discomfort. She rates the discomfort as NRS 5, with some relief after sleeping in an upright position in an armchair but worsening again shortly before contacting emergency services.

**A:** Airway patent.
**B:** Breath sounds clear bilaterally. Mild dyspnea noted.
**C:** Regular, full radial pulse. Skin normal. ECG demonstrates sinus rhythm with T-wave inversions in leads II, aVF, and V4–V6.
**D:** Alert and oriented (RLS 1).
**E:** Reports chronic musculoskeletal pain related to fibromyalgia, with symptom fluctuation. Able to ambulate to the ambulance independently but requests support due to weakness in the legs.

*Vital signs*

RR: 19
SpO₂: 97%
Pulse: 96 bpm HR: 96 bpm
BP: 135/75 mmHg
BG: 6.1 mmol/L
Temp: 36.1 °C
Pain: NRS 5/10

**Case 7**

Male, 30 years old.

*Situation*
The patient called an ambulance during the night due to sudden onset of back pain. He awoke when turning over in bed and experienced acute, cramping, and sharp pain in his back, predominantly in the lower back. Minimal movement provokes pain, whereas the pain resolves completely when he remains still in a supine position.

*Background*
Previously healthy. Employed as a plumber. The patient reports lifting a heavy refrigerator with a friend the previous day but denies sustaining any specific injury at that time.

Current status

**A:** Airway patent.
**B:** Normal breathing. Deep inspiration exacerbates back pain.
**C:** Regular radial pulse.
**D:** Alert and oriented (RLS 1). Distal neurological status intact. No sensory deficits, including absence of saddle anesthesia.
**E:** Afebrile. No visible external injuries. Palpation of the back is tolerated but provokes pain, described primarily as cramping in nature. Pain is rated as NRS 10 during acute episodes but absent when the patient remains completely still. No radiation of pain. The patient is lying supine in bed and is unable to get up.

The patient received an intramuscular injection of ketorolac without immediate effect. Following an intramuscular injection of morphine, the patient was able to get out of bed with difficulty and ambulate independently. He required support by holding onto walls or the examiner and intermittently stopped due to sharp back pain.

*Vital signs*

RR: 14
SpO₂: 100%
Pulse: 64 bpm
BP: 110/80 mmHg

Alert
Temp: 37.0 °C
Pain: NRS 7/10

**Case 8**

Female, 75 years old.

*Situation*
You are dispatched to a woman with breathing difficulties. She has a known history of COPD, and her respiratory symptoms have progressively worsened over the past few days. She lives in an apartment and is seated in an armchair upon your arrival, breathing heavily.

*Background*
Stage 3 COPD. History of heart failure and hypertension. Uses multiple inhalers daily. Current medications include ramipril and furosemide. Stopped smoking 10 years ago. Uses a walker when outdoors.

*Current status*
The patient reports increasing respiratory difficulty over the past several days. She has been sleeping in an upright position in her armchair. She experiences severe dyspnea on exertion and prefers to remain seated upright. She is able to walk only a few steps before needing to stop due to shortness of breath.

**A:** Airway patent.
**B:** Conversational dyspnea. Shallow breathing with prolonged expiration. Regular use of a PEEP device. She has taken multiple inhalations without perceived improvement. Lips mildly cyanotic. Baseline oxygen saturation reportedly 93%; currently 89% on room air. Use of accessory muscles noted. Bilateral expiratory wheezes on auscultation.
**C:** Regular radial pulse. Mild jugular venous distension observed. ECG demonstrates left ventricular hypertrophy, known from previous records.
**D:** Awake, alert, and oriented. Responds appropriately to questions. Reports anxiety related to breathing difficulties.
**E:** Thin body habitus. Afebrile. Mild bilateral ankle edema. Sitting with legs supported on a footstool. Usually wears compression stockings but has been unable to put them on today.

*Vital signs prior to treatment:*
RR: 28, SpO₂: 89% (room air), Pulse: 100 bpm, BP: 138/77 mmHg, Temp: 36.5°C

*Vital signs after inhalation with salbutamol and ipratropium bromide, followed by administration of 1 liter of oxygen via face mask:*

RR: 25, SpO₂: 91%, Pulse: 103 bpm, HR: 100 bpm, BP: 138/77 mmHg, Alert, BG: 7.9 mmol/l, Temp: 36.5°C

**Case 9**

Male, 87 years old.

*Situation*Early one morning, an ambulance was dispatched to an elderly man with breathing difficulties and generalized weakness. His wife called for an ambulance after the man ended up the floor. Upon arrival, the patient appeared confused and difficult to communicate with.

*Background*History of atrial fibrillation, hypertension, and previous sepsis. Current medications include apixaban, metoprolol, candesartan, and finasteride. Typically ambulates with a cane. Uses a hearing aid. Baseline cognitive function is reported as adequate. The couple manages at home, although the wife handles most practical matters.

*Current status*The patient’s wife provides most of the medical history, as the patient is minimally communicative. During the assessment, he appears restless, picking at objects and scanning the environment; his hands demonstrate a mild tremor. Symptoms began the previous evening. The wife reports that he appeared short of breath and more withdrawn than usual. Overnight, he rose multiple times to use the bathroom and was restless in bed. He has not eaten breakfast or taken his morning medications. In the morning, his wife observed him slump and nearly sit down on the floor. She had difficulty assisting him into a chair and did not observe any injury, as the fall was slow. The patient has attempted to stand several times but has repeatedly sat back down. He does not rise when prompted and, when assisted, sits down again as if his legs are unable to support him.

**A:** Airway patent.
**B:** Crackles auscultated over the left lung; breath sounds clear on the right. Rapid, shallow respirations.
**C:** Irregular radial pulse. Skin slightly pale. Capillary refill time is approximately 4 seconds in the fingers. Hands and feet cool to touch. No increased pedal edema.
**D:** Moves all extremities. Responds with only a few words but without apparent dysarthria. No facial asymmetry. Smiles when prompted. RLS 2. Pupils equal in size and reactive to light.
**E:** Hands and feet pale. Skin dry. Febrile. No visible injuries to the body or head. No new wounds or rashes observed.

*Vital signs*

RR: 27, SpO₂: 89%, Pulse: 103 bpm, BP: 140/70 mmHg, Confused,
Temp: 38.1 °C

**Case 10.**

Male, 84 years old.

*Situation*
The patient experienced sudden onset of chest pain while walking his dog late in the evening.

*Background*
History of hypertension. Status post cholecystectomy 10 years ago. Currently takes an ACE inhibitor. Generally in good health. Lives with his wife in a house, which he maintains independently.

*Current status*
The pain is present regardless of position (sitting, lying down, or standing) but worsens with movement. The patient describes the pain as sharp and stabbing in nature, localized to the chest with radiation toward the left side of the chest and left shoulder blade. The pain is constant. He currently rates the pain as NRS 6, with intermittent exacerbations up to NRS 8–9. The pain has been ongoing for approximately 30 minutes. He reports no prior episodes of similar pain. He denies any trauma and cannot identify any precipitating activity or injury.

**A:** Airway patent.
**B:** Breath sounds clear bilaterally, normal breathing.
**C:** Regular radial pulse. ECG shows normal sinus rhythm. Blood pressure equal in both arms.
**D:** Alert and oriented (RLS 1).
**E:** Skin slightly moist and somewhat pale. The patient is able to ambulate to the ambulance independently.

Vital signs

RR: 24

SpO₂ 96%

Pulse: 78 bpm

HR: 78 bpm

BP: 150/90 mmHg

BG: 6.5 mmol/l

Alert

Temp: 36.1 °C

Pain: NRS 8/10

**Case 11.**

Female, 63 years old.

*Situation*
The patient contacted emergency medical services late in the evening due to leg pain and difficulty ambulating. Upon arrival, she was seated in an armchair in an untidy apartment. She reports having remained seated there for the past several days, except for short trips to the bathroom or kitchen, which she describes as very difficult. She states that she has lacked the energy to contact her primary care physician.

*Background*
History of fibromyalgia and exhaustion-related depression. On disability pension. Current medications include pregabalin, acetaminophen, and duloxetine.

*Current status*
Over the past several weeks, the patient has experienced increasing fatigue and diffuse body pain, along with progressively worsening sleep disturbances. During the past week, she has developed increasing pain in both legs, resulting in growing difficulty with ambulation. She reports that touching her legs is painful and that her skin feels tender and painful to touch. She is able to stand but quickly becomes fatigued and prefers to sit down again. During the daytime, she has been able to move around her apartment independently.

**A:** Airway patent.
**B:** Breath sounds clear bilaterally.
**C:** Normal skin color. Regular radial pulse. ECG shows no arrhythmias or signs of ischemia.
**D:** ACUTE test negative. Alert and oriented (RLS 1). Pupils equal and reactive to light.
**E:** Afebrile. Skin intact. No visible swelling of the legs; distal circulation and sensation intact.

The patient lives alone in an apartment located one flight of stairs above the entrance level. She has a daughter who lives in the same city and typically assists her with grocery shopping.

*Vital signs*

RR: 19

SpO₂: 97%

Pulse: 77 bpm HR: 77 bpm

BP: 115/70 mmHg

Alert

Temp: 36.1

BG: 5.7 mmol/l

**Case 12**

*Female, 52 years old.*

*Situation*A woman experienced sudden onset of vertigo earlier in the morning. She reports feeling unwell since the morning hours and then acutely developed vertigo accompanied by nausea. She has vomited multiple times prior to ambulance arrival, approximately four hours after symptom onset. Upon arrival, she is lying supine on the bathroom floor. She prefers to remain still with her eyes closed, as head movement significantly worsens the vertigo.

*Background*
Previously healthy. Employed full-time in an office setting. Reports a recent upper respiratory infection with low-grade fever during the preceding week.

*Current status*

**A:** Airway patent.
**B:** Normal breathing.
**C:** Regular radial pulse. ECG demonstrates sinus rhythm without abnormalities.
**D:** FAST test without abnormal findings. The vertigo developed over a few minutes and has been persistent since onset. Head movement exacerbates the vertigo, although symptoms remain present even when she is still. She describes a spinning (“carousel”) sensation. She was able to ambulate to the bathroom with support from her husband but felt she would fall to the right without assistance. Pupils equal in size and reactive to light. On extraocular movement testing, left-beating nystagmus is observed, with difficulty maintaining visual fixation. Denies head trauma or headache.
**E:** Afebrile.

*Vital signs*

RR: 18
SpO₂: 99%
Pulse: 75 bpm HR: 75 bpm
BP: 130/80 mmHg
BG: 6.3 mmol/L

Alert

Temp: 37.0 °C
Pain: 0

**Case 13**

Male, 80 years old.

*Situation*
You are dispatched to a man residing in a nursing home due to breathing difficulties. Nursing staff report that the patient appeared more agitated and fatigued than usual when they entered his room in the morning. Upon arrival, the patient is still in bed with his head slightly elevated.

*Background*
History of Alzheimer’s disease. The patient is typically talkative and cheerful but lacks short-term memory. No other known medical conditions. He usually ambulates independently within the ward, although unsteadily. The patient recently had an upper respiratory infection and has been bedridden during the past week. He has been afebrile for the past few days. Several staff members and residents at the facility have recently been ill with a viral infection.

*Current status*
The patient appeared to be recovering from his recent cold a few days ago but deteriorated again this morning.

**A:** Airway patent. Audible upper airway wheezing when coughing.
**B:** Auscultation reveals coarse crackles over the left lung. Breath sounds are diminished bilaterally.
**C:** Regular radial pulse. ECG without abnormalities. Capillary refill approximately 3 seconds when assessed centrally over the sternum.
**D:** Emergency neurological screening cannot be performed. Moves all extremities spontaneously. RLS 2; somnolent. The patient looks up when called upon and by tactile stimulation. Responds with a few words when addressed but quickly falls back asleep. Pupils equal in size and reactive to light.
E: Afebrile. Effective cough. Skin somewhat pale but otherwise intact.

*Vital signs*

RR: 24
SpO₂: 92%
Pulse: 75 bpm HF: 75 bpm
BP: 120/65 mmHg

Voice

BG: 8.0 mmol/l
Temp: 36.0 °C

**Case 14**

Male, 75 years old.

*Situation*
You are dispatched to a man following a syncopal episode. Upon arrival, the patient is awake and alert and reports no prodromal symptoms prior to the event and is unsure how it occurred. His daughter, who witnessed the episode, states that the patient was standing in the kitchen when he suddenly collapsed. He was unconscious for approximately 30 seconds before regaining consciousness. During the episode, she observed mild body twitching, pallor, and heavy, snoring respirations.

*Background*
History of hypertension treated with medication. Current medication includes bendroflumethiazide (Salures).

*Current status*

**A:** Airway patent.
**B:** Normal breathing.
**C:** Regular radial pulse. Skin color normal. ECG demonstrates a left bundle branch block, previously unknown to the patient. Not considered relevant for percutaneous coronary intervention (PCI). Blood pressure equal in both arms.
**D:** Alert and oriented (RLS 1). No focal neurological deficits. Reports transient dizziness following the collapse, which resolved spontaneously.
**E:** No signs of traumatic injury related to the fall. Denies pain. Able to ambulate independently.

*Vital signs*

RR: 16
SpO₂: 98%
Pulse: 68 bpm HR: 68 bpm
BP: 125/80 mmHg

Alert
BG: 5.5 mmol/L
Temperature: 37.2 °C

**Case 15**

Female, 90 years old.

*Situation*
Home care services contacted EMS for a woman who had fallen and was lying on the floor, unable to get up. The patient, who uses a walker, lost her balance and fell backward, pulling the walker with her. She landed in a seated position and complains of pain in her right hip and leg.

*Background*
History of osteoporosis and vertebral compression fractures. Previous distal radius fracture related to an earlier fall. Hypertension and prior transient ischemic attack. Current medications include acetylsalicylic acid, candesartan, buprenorphine transdermal patch, acetaminophen, vitamin B12, vitamin D, and calcium supplementation.

*Current status*

**A:** Airway patent.
**B:** Breath sounds clear bilaterally.
**C:** Radial pulse regular with occasional missed beats. Skin color normal.
**D:** Alert and oriented (RLS 1). No head strike reported. Pupils equal in size and reactive to light. Moves all extremities; movement of the right leg is painful. Moans loudly with attempts at mobilization. Distal neurovascular status intact. Marked tenderness over the right hip, with slight shortening and external rotation of the right lower extremity.
**E:** Erythema over the right thigh after prolonged time on the floor. Generalized tenderness over the lower back, consistent with baseline. No other visible injuries.

The patient responded well to intravenous analgesia and reported acceptable pain control when the leg was kept immobile, allowing transfer to the stretcher.

*Vital signs*

RR: 21
SpO₂: 95%
Pulse: 70 bpm
BP: 120/65 mmHg

Alert
Temp: 36.1 °C
Pain: NRS 3/10

**Case 16**

Female, 66 years old.

*Situation*
A woman has been experiencing right-sided abdominal pain for the past three days, with progressive worsening. She reports associated nausea.

*Background*
History of type 2 diabetes mellitus treated with metformin. Hypertension treated with an ACE inhibitor.

*Current status*
Gradual onset three days prior. Initially, the pain was mild, dull, and intermittent, with temporary relief from acetaminophen. Today, the pain has become constant. The pain is primarily localized to the right upper quadrant beneath the costal margin and is also tender in the epigastric region on palpation. She took acetaminophen several hours prior without relief. The pain is exacerbated by deep inspiration. Murphy’s sign is positive and tender. She describes a dull pain radiating to the right side of the back, with intermittent worsening. She reports nausea, rated as NRS 5.

**A:** Airway patent.
**B:** Normal breathing; breath sounds clear bilaterally.
**C:** Regular radial pulse. ECG without abnormalities.
**D:** Alert and oriented (RLS 1).
**E:** Skin normal.

The patient reports loose stools during the day but denies vomiting. She has had reduced oral intake. She is able to walk to the ambulance independently. The patient received an intramuscular injection of ketorolac with good effect and subsequently reported pain intensity of NRS 4.

*Vital signs*

RR: 20
SpO₂: 96%
Pulse: 88 bpm HR: 88 bpm
BP: 140/80 mmHg

Alert

BG: 10.5 mmol/l
Temp: 37.8 °C
Pain: NRS 4/10

**Case 17.**

Male, 25 years old.

*Situation*
EMS were called by other road users who witnessed a high-speed motor vehicle collision in which a car struck the guardrail, skidded, and came to rest in a ditch on the opposite side of the road. The driver, a 25-year-old male, was the sole occupant of the vehicle. Airbags deployed. The vehicle, approximately 10 years old, sustained significant front-end damage, and the left front tire was missing. Upon arrival, the patient remained seated in the vehicle with his seatbelt fastened, in accordance with instructions from emergency dispatch. He appeared shaken. On initial assessment, he reported neck pain with tenderness to palpation and movement. He was otherwise neurologically intact and also complained of pain in the left shoulder blade.

*Background*
Previously healthy. No known medical conditions.

*Current status*
The ambulance arrived approximately 25 minutes after the collision. On examination, the cervical paraspinal muscles were tender to palpation, with no midline vertebral tenderness. The patient described sharp pain over the left shoulder blade with movement, without radiation. Pain on palpation of the neck was rated as NRS 4. Pain in the left shoulder blade was rated as NRS 6 during movement and absent at rest.

**A:** Airway patent; speaking clearly without difficulty.
**B:** Breath sounds equal bilaterally with symmetrical chest expansion. Pain from the shoulder blade is elicited during deep inspiration.
**C:** Radial pulse palpable and regular. No visible external bleeding.
**D:** Pupils equal and reactive to light. Neurologically intact.
**E:** Skin color normal. No visible external injuries.

*Vital signs*

RR: 18
SpO₂: 99%
Pulse: 91 bpm
BP: 120/80 mmHg

Alert
Temp: 37.0 °C
Pain: NRS 6/10

**Case 18.**

Female, 60 years old.

*Situation*
A woman fell off her bicycle while riding home after dinner with friends. She was wearing a helmet but struck her head on the asphalt and, according to witnesses, experienced a brief loss of consciousness lasting approximately 15 seconds. Upon arrival, she is seated on a park bench, leaning against a friend. She has been up and walking. She smells of alcohol and reports nausea, neck stiffness, and a mild headache.

*Background*
History of hypertension treated with an ACE inhibitor. Takes acetylsalicylic acid for an unclear indication.

*Current status*

**A:** Airway patent; speaking without difficulty.
**B:** Symmetrical chest expansion with clear breath sounds bilaterally.
**C:** Regular radial pulse. No major visible external bleeding.
**D:** Awake and alert but intoxicated, with mild slurring of speech. She does not recall the fall itself but remembers cycling home from dinner. Pupils slightly enlarged but equal in size and reactive to light. No focal neurological deficits. No midline cervical or spinal tenderness on palpation; however, she reports neck stiffness with movement. She denies neck pain but complains of a mild headache.
**E:** Abrasions to the right knee, right arm, and chin. The helmet shows superficial damage on the right side. No palpable scalp hematoma.

*Vital signs*

RR: 20
SpO₂: 96%
Pulse: 83 bpm
BP: 120/65 mmHg

Alert

BG: 7.5 mmol/l
Temp: 36.5 °C
Pain: NRS 3/10

**Case 19.**

Female, 85 years old.

*Situation*
You are dispatched to an elderly woman at the request of her son because she is unable to get out of bed. According to both the patient and her son, her health has gradually declined over several months, with reduced appetite, weight loss, and multiple falls. She feels generally weak and fatigued and sleeps for much of the day. Her son also reports that she has become quieter and appears to have worsening memory compared to a few months ago.

*Background*
History of hypertension treated with medication and a prior stroke without residual deficits 10 years ago. Previously consumed large amounts of alcohol but has abstained since her stroke. Current medications include acetylsalicylic acid, amlodipine, and atorvastatin. Uses nutritional supplements. Receives home care assistance for cleaning and meal delivery. Ambulates with a walker at baseline.

*Current status*

**A:** Airway patent.
**B:** Breath sounds clear apically. Breath sounds somewhat diminished at the lung bases when supine.
**C:** Radial pulse palpable and regular with occasional missed beats. ECG without abnormalities.
**D:** Able to lift both arms. Grip strength reduced bilaterally. Lower extremity strength reduced symmetrically. No remarks on speech with normal facial motor function. Pupils slightly small but equal in size and reactive to light. RLS 1. Oriented to person and place but not to day or year. Unable to recall activities, food intake, or time of day.
**E:** No new wounds noted. Several older bruises present. Coated tongue. Afebrile. With assistance from two people, the woman is able to get out of bed and stand briefly but is unable to ambulate and immediately wishes to sit down. Denies pain.

The patient initially waves away assistance and expresses a desire to return to sleep but agrees to examination.

*Vital signs*

RR: 24
SpO₂: 93%
Pulse: 59 bpm
BP: 120/65 mmHg

Confused

BG: 9.6 mmol/l
Temp: 36.2 °C
Pain: 0

**Case 20.**

Female, 29 years old.

*Situation*
You respond to a woman who reports back pain, fever, and vomiting.

*Background*
Uses oral contraceptives. History of two urinary tract infections during the past year. Otherwise previously healthy.

*Current status*
The patient has felt unwell for two days and has had fever since the previous day. She reports sharp pain in the lower back, accompanied by recurrent chills. There is tenderness to palpation over the right lower back in the costovertebral area. The pain has increased since yesterday and radiates toward the right flank. She denies any trauma. She took acetaminophen and ibuprofen several hours earlier without noticeable relief. She rates the pain as NRS 9. The patient reports nausea and has vomited twice within the past four hours. She also reports urinary frequency associated with a burning sensation during urination.

**A:** Airway patent.
**B:** Breath sounds clear bilaterally.
**C:** Radial pulse strong, regular, and tachycardic.
**D:** AKUT test without abnormal findings. Alert and oriented (RLS 1).
**E:** Skin warm with flushed facial appearance. Appears to be in significant pain, has difficulty remaining still in bed, and expresses concern. Able to stand and ambulate independently but is visibly affected by fever and pain and reports generalized weakness.

*Vital signs*

RR: 22
SpO₂: 98%
Pulse: 116 bpm
BP: 100/52 mmHg

Alert

BG: 7.3 mmol/l
Temp: 39.0 °C
Pain: NRS 9/10

**Case 21.**

Male, 81 years old.

*Situation*
EMS were contacted by home care staff after the patient vomited material described as coffee-ground–like. Upon arrival, the patient is found seated in a wheelchair and appears frail. Dark vomitus is present around his mouth and in his beard. Home care staff present a plastic bag containing remnants of the vomitus retrieved from the trash; the vomitus is dark brown in color and watery in consistency.

*Background*
History of myocardial infarction one month ago, treated with PCI. Current medications include metoprolol, prasugrel, acetylsalicylic acid, and omeprazole. According to home care staff, the patient has complained of mild abdominal discomfort over the past several days.

*Current status*
The patient reports ongoing abdominal pain that began several days ago, although he is unable to specify the exact onset. He denies nausea. The abdomen appears scaphoid. Palpation reveals tenderness in the epigastric region, while the remainder of the abdomen is soft and non-tender. He denies radiation of pain and is unable to state whether the pain is affected by position. He reports reduced appetite and states that the abdominal discomfort worsens with oral intake. He has difficulty characterizing the pain but localizes it to the epigastrium. Pain intensity is reported as NRS 4. He is uncertain whether he has had melena or bloody stools. He has not eaten anything today and has only consumed a small amount of juice.

**A:** Airway patent.
**B:** Normal breathing with clear breath sounds bilaterally.
**C:** Radial pulse weak but regular. Skin pale and slightly moist. Hands cold to touch. Capillary refill approximately 3.5 seconds when assessed centrally over the sternum. ECG without abnormalities.
**D:** Alert and oriented (RLS 1).
**E:** Afebrile.

*Vital signs*

RR: 20
SpO₂: 95%
Pulse: 62 bpm HR: 62 bpm
BP: 120/70 mmHg

Alert
Temp: 36.9 °C
Pain: NRS 4/10

**Case 22.**

Male, 66 years old.

*Situation*
You are dispatched to a man at the request of his wife, who suspects hypoglycemia due to abnormal behavior. She recognizes the behavior from a previous episode of low blood glucose. She is unable to administer oral sugar safely, as the patient is displaying aggressive behavior.

*Background*
History of type 2 diabetes mellitus, angina pectoris, and hypertension. Treated with metformin, sitagliptin, nitroglycerin as needed, and ramipril. Uses both long-acting and short-acting insulin.

*Current status*
According to his wife, the patient took his morning medications, including mealtime insulin. He then went for an unusually long walk with the family dog and subsequently spent additional time gardening outdoors. Upon returning indoors, he appeared quieter than usual and is now seated in the kitchen staring straight ahead. He has not responded verbally when spoken to and has only stared at his wife. When she attempted to touch him and offer something sweet, he waved his arms and behaved threateningly.

You and your colleague were able to administer honey orally and obtained a capillary BG measurement, which was 2.5 mmol/L. Shortly thereafter, the man becomes more alert and able to communicate. He was administered an additional dose of 30% glucose intravenously and given a glass of milk. A repeat blood glucose measurement showed 4.0 mmol/L. The patient reports uncertainty regarding whether he administered the correct insulin dose before breakfast and states that he now feels back to normal.

**A:** Airway patent.
**B:** Breath sounds clear bilaterally.
**C:** Regular radial pulse.
**D:** Following intravenous glucose administration, awake and alert, oriented to person, place, and time.
**E:** Skin normal.

*Vital signs*

RR: 14
SpO₂: 98%
Pulse: 60 bpm
BP: 130/70 mmHg

Alert

BG: 4.0 mmol/l
Temp: 36.1 °C

**Case 23.**

Female, 30 years old.

*Situation:*
You are dispatched to a woman in her 30s who has been found unconscious outdoors by passers-by. Upon arrival, the patient is found lying on a lawn in a park, positioned on her side. She does not respond to verbal stimuli.

*Background:*
Unknown.

*Current status*
No bystanders are able to identify the patient. It is a warm summer evening. The patient is wearing pants, a shirt, and a jacket covering her body.

**A:** Airway patent.
**B:** Normal breathing with clear breath sounds bilaterally.
**C:** Regular radial pulse.
**D:** Unresponsive to verbal stimuli. On painful stimulation, the patient demonstrates slight facial grimacing and minimal shoulder shrugging but no purposeful or defensive movements. Level of consciousness assessed as RLS 5–6. Pupils slightly dilated, equal in size, and symmetrically reactive to light. Gaze appears slightly upward.
**E:** No visible injuries to the body or head. Not obviously hypothermic. Clothing dry. No visible puncture marks. No odor of alcohol or other unusual substances detected.

*Vital signs*

RR: 12
SpO₂: 98%
Pulse: 91
BP: 110/80 mmHg

Unresponsive

BG: 5.0 mmol/l
Temp: 35.9 °C

**Case 24.**

Female, 67 years old.

*Situation*
EMS were contacted by the patient’s daughter after a telephone conversation during which the patient sounded confused and was speaking incoherently. The daughter became concerned due to the patient’s known history of Addison’s disease. Upon arrival, the patient is found lying in bed. She is warm to the touch and demonstrates mild slurring of speech. She has vomited into a bucket. In the kitchen, an unused ampoule of Solu-Cortef with an expired date is found.

*Background*
Known history of Addison’s disease. Medications found on scene include hydrocortisone tablets, Solu-Cortef, levothyroxine, and ramipril.

*Current status*

**A:** Airway patent.
**B:** Respirations somewhat rapid and shallow. Breath sounds clear bilaterally.
**C:** Radial pulse weak and tachycardic.
**D:** Responds to verbal stimuli but appears fatigued and confused. Level of consciousness assessed as RLS 2. Moves all extremities without limitation. Pupils equal in size and symmetrically reactive to light.
**E:** Notable hyperpigmentation, particularly of the palms and perioral area. Skin warm and dry. The patient reports headache but is unable to quantify the intensity, describing it as moderate.

When assisted to sit on the edge of the bed, the patient becomes dizzy and requires lying down again. She is transferred directly from the bed to the stretcher.

*Vital signs*

RR: 24
SpO₂: 96%
Pulse: 109
BP: 105/65 mmHg

Alert

BG: 4.4 mmol/l
Temp: 38.7 °C

**Case 25.**

Male, 41 years old.

*Situation*
You are dispatched to an apartment after a man contacted EMS due to concerns related to his substance use. He reports having used amphetamines for several consecutive days and has developed palpitations and anxiety earlier today. He meets the ambulance crew outside the building.

*Background*
History of long-term substance use disorder with intermittent drug use. Over the past week, he has used large amounts of amphetamine, both intravenously and orally. Earlier today, he also consumed a few beers. He is a smoker and has chronic hepatitis C.

*Current status*

**A:** Airway patent.
**B:** Normal breathing.
**C:** Radial pulse palpable. Skin color normal; skin dry. ECG demonstrates sinus rhythm with tachycardia.
**D:** Alert and oriented (RLS 1), mildly agitated. Pupils dilated but reactive to light.
**E:** Visible injection marks on both arms.

The patient reports that his last amphetamine use was approximately two hours prior to assessment. He expresses significant anxiety after experiencing sudden, forceful palpitations and a sensation that he was about to die.

*Vital signs*

RR: 14
SpO₂: 97%
Pulse: 110
BP: 150/90 mmHg

Alert

BG: 6.1mmol/l
Temp: 37.5 °C

**Case 26.**

Female, 65 years old.

*Situation*
The woman experienced a sudden onset of severe headache while showering. Upon arrival, she is found lying on a sofa with her eyes closed, holding her head.

*Background*
Previously healthy. History of migraine headaches, which she reports are distinctly different from the current headache.

*Current status*
The patient reports that the pain began suddenly and with high intensity. She describes a throbbing headache, predominantly located in the occipital region. She reports neck stiffness and difficulty extending her neck to look upward. She prefers to keep her eyes closed. She states that this is the worst headache she has ever experienced and rates the pain as NRS 10. She denies dizziness but reports nausea. She is able to stand but finds it difficult to speak due to the severity of the pain. She denies any head trauma.

**A:** Airway patent.
**B:** Normal breathing.
**C:** Regular radial pulse.
**D:** Alert and oriented (RLS 1). No focal neurological deficits. Pupils normal in size and reactive to light. Photophobia present.
**E:** Neck stiffness noted, with inability to flex the neck to bring the chin to the chest. No petechiae. Afebrile.

*Vital signs*
RR: 17
SpO₂: 98%
Pulse: 80 bpm
BP: 140/80 mmHg

Alert
Temp: 37.5°C

Pain: NRS 10/10

**Case 27.**

Female, 32 years old.

*Situation*
A pregnant woman in her third trimester contacted EMS due to sudden onset of abdominal pain.

*Background*
Previously healthy. Currently 34 weeks pregnant. Lives alone at home with a 2-year-old child. The pregnancy has been uncomplicated to date.

*Current status*
The patient awoke during the night with severe abdominal pain, which she does not recognize as labor pain or uterine contractions. The ambulance arrived approximately 45 minutes after symptom onset. The pain has been constant since onset.

**A:** Airway patent.
**B:** Normal breathing with clear breath sounds bilaterally.
**C:** Radial pulse palpable and tachycardic. The patient denies vaginal bleeding. Skin dry with normal color. She reports having broken out in a cold sweat at the onset of pain.
**D:** Alert and oriented (RLS 1).
**E:** Abdomen firm and tender to palpation. The patient reports perceiving fetal movements. Afebrile. No bruising or visible external injuries. She rates the pain as NRS 7 and describes it as sharp, localized to the lower abdomen.

The patient is packing a bag and wishes to wait for her mother to arrive to care for her 2-year-old child. She is able to ambulate independently but intermittently stops, moans due to pain, and holds her abdomen. She denies any abdominal trauma.

*Vital signs*
RR: 25
SpO₂: 100%
Pulse: 90 bpm
BP: 127/100 mmHg

Alert

BG: 5.0 mmol/l

Temp: 37.4°C

Pain: NRS 7/10

**Case 28.**
Male, 66 years old.

*Situation*
A man experienced an allergic reaction after accidentally ingesting kiwi at a social gathering, to which he has a known allergy. He reports that the reaction is more severe than his usual allergic responses and includes breathing discomfort and widespread hives.

*Background*
Known allergy to kiwi and pineapple. Typically experiences oral tingling and urticaria with exposure. Otherwise healthy.

*Current status*
Upon arrival, the patient is seated. He reports ingesting kiwi approximately one hour prior, with symptom onset about 30 minutes thereafter. He has pruritic urticaria involving the torso, back, and thighs. He complains of a stinging sensation in the mouth and on the tongue. He was administered 5 mg of desloratadine at the onset of symptoms, after which the urticaria has partially subsided.

**A:** Airway patent. Reports stinging sensation in the mouth.
**B:** Breath sounds clear bilaterally. Respirations appear unlabored, although the patient reports subjective difficulty breathing.
**C:** Regular radial pulse.
**D:** Alert and oriented (RLS 1).
**E:** Marked urticaria over the body. Facial erythema present. No visible angioedema. No visible swelling of the tongue or throat.

The patient is ambulatory without assistance.

*Vital signs*
RR: 22
SpO₂: 98%
Pulse: 85 bpm
BP: 140/90 mmHg

Alert

Temp: 36.8°C

**Case 29.**

Male, 82 years old.

*Situation*

A man contacted EMS due to episodic dizziness.

*Background*
History of hypertension and hyperlipidemia. Treated with an ACE inhibitor and a statin.

*Current status*
Approximately two hours prior to assessment, the patient experienced sudden onset of dizziness and was forced to sit down. He describes the sensation as dizziness “inside his head” and reports feeling unsteady and near-syncope shortly before calling the ambulance. The dizziness improved with rest but recurred upon standing and ambulating. He denies chest pain and headache.

**A:** Airway patent.
**B:** Breath sounds clear bilaterally; normal breathing.
**C:** Radial pulse slow and regular. ECG demonstrates bradycardia with a heart rate ranging from 39 to 50 beats per minute. Intermittent non-conducted QRS complexes are observed, consistent with second-degree atrioventricular block, Mobitz type II. Skin dry at rest; however, during transfer to the ambulance, the patient becomes pale and mildly diaphoretic. He then requires support due to weakness, nausea, and dizziness.
**D:** No focal neurological deficits. Alert and oriented (RLS 1). Pupils normal in size and reactive to light.
**E:** No additional findings.

The patient improves again with rest and reports feeling relatively well once lying supine on the stretcher.

Vital signs
RR: 20
SpO₂: 97%
Pulse: 41 bpm HR: 41 bpm
BP: 141/66 mmHg

Alert

Temp: 36.3°C

**Case 30.**

Female, 60 years old.

*Situation*
A woman reports feeling faint when standing up. She has experienced vaginal bleeding for the past three months. She has delayed seeking medical care, hoping the bleeding would resolve spontaneously. However, she became alarmed after feeling increasingly weak and experiencing near-blackout of vision when attempting to stand up from the sofa. Upon arrival, she reports feeling more alert and is able to ambulate independently without dizziness or unsteadiness.

*Background*
History of hypertension treated with medication; currently takes losartan. Menopause at age 50, with no vaginal bleeding since that time until the current episode. Reports unintentional weight loss of approximately 10 kg over the past two months despite unchanged appetite. Lives alone in a townhouse. No children. Employed as an economist.

*Current status*
The patient reports that the vaginal bleeding was initially mild but has progressively worsened.

**A:** Airway patent.
**B:** Normal breathing.
**C:** Radial pulse somewhat weak but regular. Skin dry and somewhat pale. Reports using approximately 3–4 sanitary pads per day. The bleeding is not malodorous.
**D:** Alert and oriented (RLS 1).
**E:** Reports intermittent pelvic pain described as a dull, low abdominal pain resembling menstrual cramps. Currently mild (NRS 2), with episodes up to NRS 5–6 at worst. She has taken acetaminophen and ibuprofen with partial relief. The abdomen is soft, with mild tenderness to palpation just above the pubic symphysis.

*Vital signs*
RR: 20
SpO₂: 98%
Pulse: 90 bpm
BP: 115/85 mmHg

Alert

Temp: 36.5°C

Pain: NRS 2/10

**Additional file 2.**

**Discriminators**

**
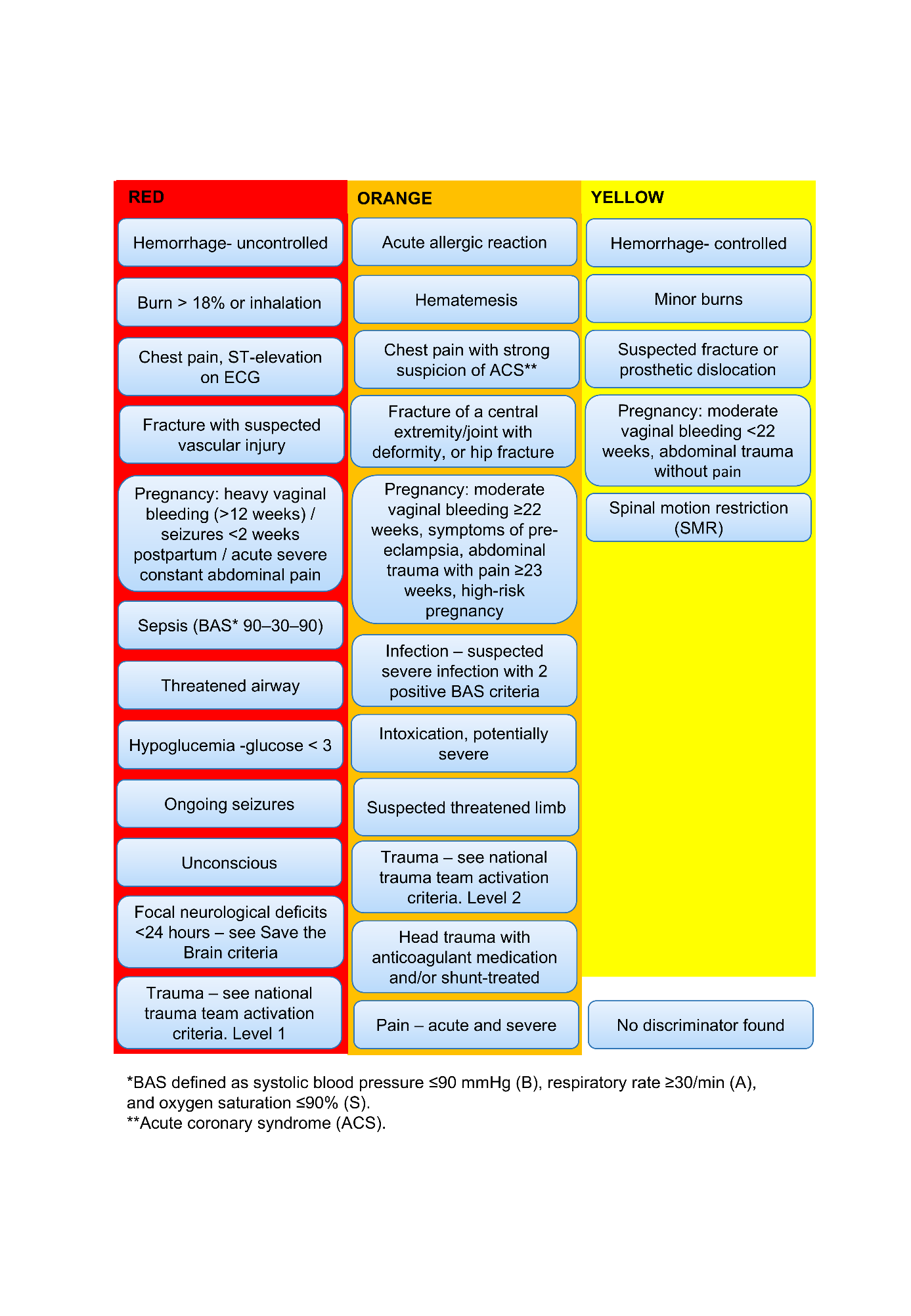
**

**Additional file 3.**

Full set of questions from questionnaire:

1. Specify whether the patient should receive 1 point for trauma: Yes/No

- Yes (adds 1 additional point to TEWS)
- No

1. Specify the mobility status you consider appropriate based on any intervention performed.

- Walking
- With help (adds 1 point in TEWS)
- Stretcher/Immobile (Adds 2 points in TEWS)

1. How many total TEWS points does the patient receive?

☐ 0 ☐ 1 ☐ 2 ☐ 3 ☐ 4 ☐ 5 ☐ 6 ☐ 7 ☐ 8 ☐ 9 ☐ 10

1. If you consider a relevant discriminator to be present, specify which one. If not, answer no.
2. Enter the total triage color.

- Green triage (0-2 points / No discriminator)
- Yellow triage (3-4 points and/or yellow discriminator)
- Orange triage (5-6 points and/or orange discriminator)
- Red triage (7 or more points and/or red discriminator)

1. If you choose to manually upgrade the patient’s triage level, specify to which color. Provide a justification if the final triage level differs from the assessment above.

**Additional file 4.**

Percent agreement on each case vignette with describing chief complaint.

| **Case vignette** | **Chief complaint** | **Percent agreement (%)** |
| --- | --- | --- |
| 1. | Chest pain | 76,5 |
| 2. | Facial numbness | 35,3 |
| 3. | Abdominal pain | 56,9 |
| 4. | Head injury | 41,2 |
| 5. | Upper arm injury | 52,9 |
| 6. | Chest discomfort | 85,3 |
| 7. | Back pain | 52,9 |
| 8. | Shortness of breath | 67,6 |
| 9. | Breathing difficulty | 79,4 |
| 10. | Chest pain | 88,2 |
| 11. | Leg pain | 85,3 |
| 12. | Vertigo | 58,8 |
| 13. | Breathing difficulty | 94,1 |
| 14. | Syncope | 32,4 |
| 15. | Hip injury | 100 |
| 16. | Abdominal pain | 52,9 |
| 17. | Neck pain after motor vehicle collision | 67,6 |
| 18. | Head injury with brief loss of consciousness | 67,6 |
| 19. | Generalized weakness | 64,7 |
| 20. | Back pain with fever and vomiting | 100 |
| 21. | Coffee-ground vomiting | 82,4 |
| 22. | Altered behavior | 85,3 |
| 23. | Unconsciousness | 94,1 |
| 24. | Altered mental status | 100 |
| 25. | Palpitations and anxiety | 52,9 |
| 26. | Sudden onset headache | 64,7 |
| 27. | Sudden abdominal pain in late pregnancy | 58,8 |
| 28. | Allergic reaction with breathing difficulty | 76,5 |
| 29. | Episodic dizziness | 70,6 |
| 30. | Near-syncope on standing | 61,7 |
