# Supplementary figures and images for: An inter-rater reliability study of a modified version of SATS as a prehospital triage tool
Source: Scand J Trauma Resusc Emerg Med. 2026 Jun 12;34:106. doi: 10.1186/s13049-026-01648-8 (PMC13263948; doi:10.1186/s13049-026-01648-8)

**Additional file 2.**

**Discriminators**


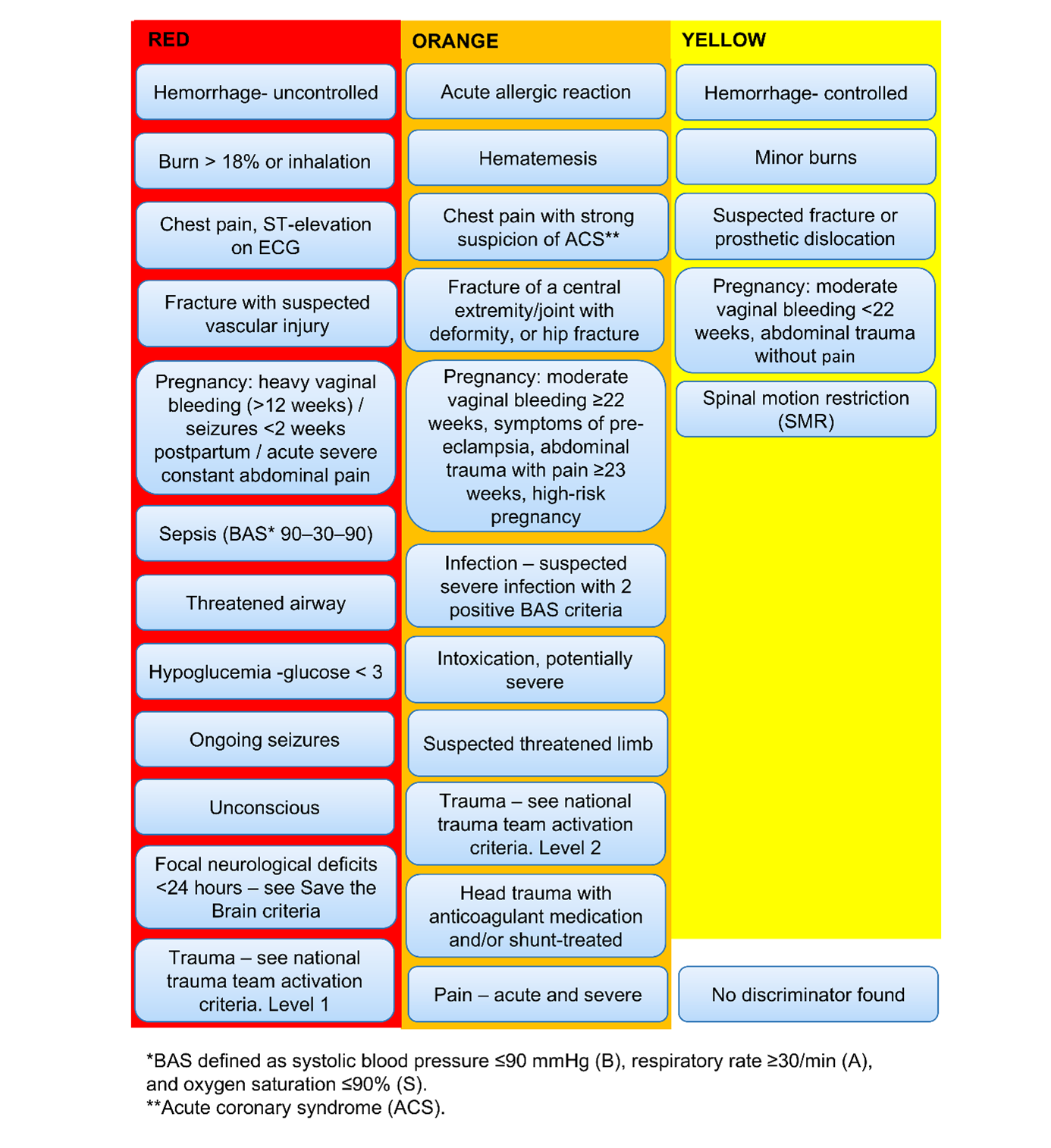

Supplement: Supplementary file 2 — Supplementary Material 2 [file 13049_2026_1648_MOESM2_ESM.docx]
