# Supplementary material for: An inter-rater reliability study of a modified version of SATS as a prehospital triage tool
Source: Scand J Trauma Resusc Emerg Med. 2026 Jun 12;34:106. doi: 10.1186/s13049-026-01648-8 (PMC13263948; doi:10.1186/s13049-026-01648-8)
Supplement: Supplementary file 3 — Supplementary Material 3 [file 13049_2026_1648_MOESM3_ESM.docx]

**Additional file 3.**

Full set of questions from questionnaire:

1. Specify whether the patient should receive 1 point for trauma: Yes/No

- Yes (adds 1 additional point to TEWS)
- No

1. Specify the mobility status you consider appropriate based on any intervention performed.

- Walking
- With help (adds 1 point in TEWS)
- Stretcher/Immobile (Adds 2 points in TEWS)

1. How many total TEWS points does the patient receive?

☐ 0 ☐ 1 ☐ 2 ☐ 3 ☐ 4 ☐ 5 ☐ 6 ☐ 7 ☐ 8 ☐ 9 ☐ 10

1. If you consider a relevant discriminator to be present, specify which one. If not, answer no.
2. Enter the total triage color.

- Green triage (0-2 points / No discriminator)
- Yellow triage (3-4 points and/or yellow discriminator)
- Orange triage (5-6 points and/or orange discriminator)
- Red triage (7 or more points and/or red discriminator)

1. If you choose to manually upgrade the patient’s triage level, specify to which color. Provide a justification if the final triage level differs from the assessment above.
