# Supplementary material for: An inter-rater reliability study of a modified version of SATS as a prehospital triage tool
Source: Scand J Trauma Resusc Emerg Med. 2026 Jun 12;34:106. doi: 10.1186/s13049-026-01648-8 (PMC13263948; doi:10.1186/s13049-026-01648-8)
Supplement: Supplementary file 4 — Supplementary Material 4 [file 13049_2026_1648_MOESM4_ESM.docx]

**Additional file 4.**

Percent agreement on each case vignette with describing chief complaint.

| **Case vignette** | **Chief complaint** | **Percent agreement (%)** |
| --- | --- | --- |
| 1. | Chest pain | 76,5 |
| 2. | Facial numbness | 35,3 |
| 3. | Abdominal pain | 56,9 |
| 4. | Head injury | 41,2 |
| 5. | Upper arm injury | 52,9 |
| 6. | Chest discomfort | 85,3 |
| 7. | Back pain | 52,9 |
| 8. | Shortness of breath | 67,6 |
| 9. | Breathing difficulty | 79,4 |
| 10. | Chest pain | 88,2 |
| 11. | Leg pain | 85,3 |
| 12. | Vertigo | 58,8 |
| 13. | Breathing difficulty | 94,1 |
| 14. | Syncope | 32,4 |
| 15. | Hip injury | 100 |
| 16. | Abdominal pain | 52,9 |
| 17. | Neck pain after motor vehicle collision | 67,6 |
| 18. | Head injury with brief loss of consciousness | 67,6 |
| 19. | Generalized weakness | 64,7 |
| 20. | Back pain with fever and vomiting | 100 |
| 21. | Coffee-ground vomiting | 82,4 |
| 22. | Altered behavior | 85,3 |
| 23. | Unconsciousness | 94,1 |
| 24. | Altered mental status | 100 |
| 25. | Palpitations and anxiety | 52,9 |
| 26. | Sudden onset headache | 64,7 |
| 27. | Sudden abdominal pain in late pregnancy | 58,8 |
| 28. | Allergic reaction with breathing difficulty | 76,5 |
| 29. | Episodic dizziness | 70,6 |
| 30. | Near-syncope on standing | 61,7 |
